# Exploring cysteine regulation in cancer cell survival with a highly specific “Lock and Key” fluorescent probe for cysteine†
†Electronic supplementary information (ESI) available: Synthesis, experimental procedures, supplemental spectra and imaging data, and ^1^H-, ^13^C-NMR, and MS spectra. See DOI: 10.1039/c9sc02618e


**DOI:** 10.1039/c9sc02618e

**Published:** 2019-09-07

**Authors:** Jing Liu, Mengxing Liu, Hongxing Zhang, Xuehong Wei, Juanjuan Wang, Ming Xian, Wei Guo

**Affiliations:** a School of Chemistry and Chemical Engineering , Shanxi University , Taiyuan 030006 , China . Email: guow@sxu.edu.cn; b Scientific Instrument Center , Shanxi University , Taiyuan 030006 , China; c Department of Chemistry , Washington State University , Pullman , WA 99164 , USA

## Abstract

To probe the regulatory roles of cysteine (Cys) in cancer cell survival, a highly selective and sensitive fluorescent Cys probe **SiR** was developed by employing a novel “lock and key” strategy, which allows Cys to be detected without any interference or probe consumption caused by the intracellular high concentration of glutathione (GSH). Using **SiR**, we confirmed that inhibiting cystine (Cys_2_) transporter system x_c_^–^ to deplete intracellular Cys is more efficient than inhibiting glutamate–cysteine ligase GCL to deplete intracellular GSH for sensitizing cancer cells to chemotherapy. Moreover, with the probe, a possible self-protection mechanism of cancer cells was indicated: when extracellular Cys sources are blocked, cancer cells could still survive by multidrug resistance protein transporter (Mrp1)-mediated export of intracellular GSH/GSSG as sources to supply intracellular Cys for resisting detrimental oxidative stress. Based on this finding, we further confirmed that abrogating the self-protection mechanism is an even more efficient strategy for sensitizing cancer cells to chemotherapy.

## Abstract 2

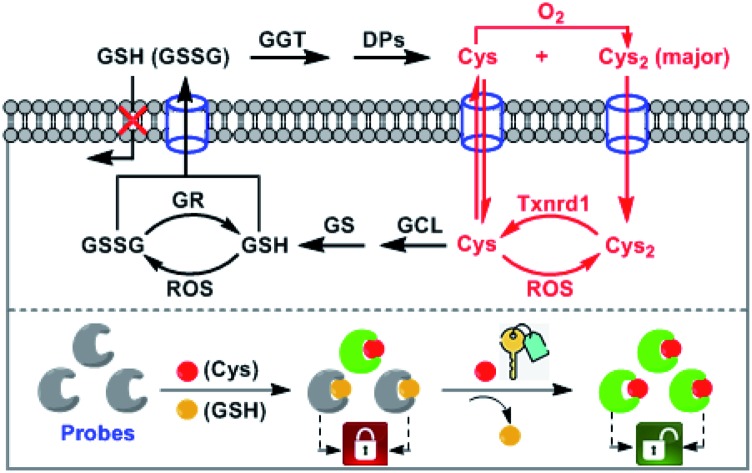
Using a highly specific “lock and key” fluorescent Cys probe, we confirmed that targeting Cys metabolism to deplete intracellular Cys is a more potent strategy to sensitize cancer cells to chemotherapies.

## Introduction

**Scheme 1:**
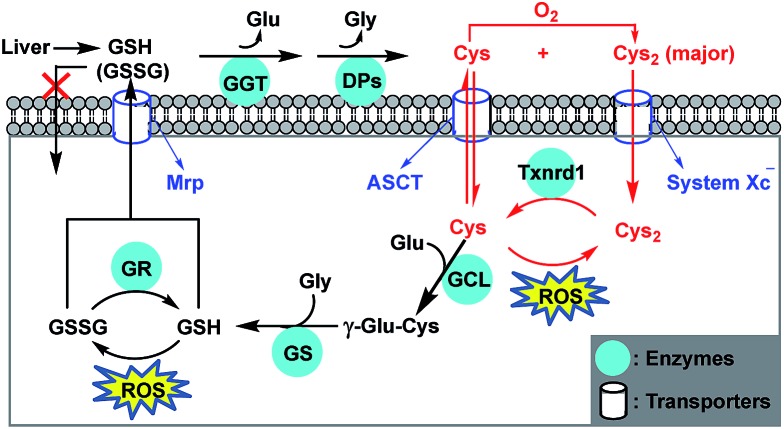
Antioxidant mechanism of GSH and Cys. Most cells cannot directly take up extracellular GSH and GSSG (oxidized form of GSH), mainly coming from liver *via* the methionine (Met)-relevant *trans*-sulfuration pathway other than diet. GSH and GSSG are cleaved extracellularly by γ-glutamyl transpeptidase (GGT) and dipeptidases (DPs) to form Cys and Cys_2_ (oxidized form of Cys). Cys is unstable and easily auto-oxidized to Cys_2_. Cys and Cys_2_ can be taken up by cells *via* ACS transporter and system x_c_^–^, respectively, and the latter dominates for most of the cells. Once inside the cell, Cys_2_ is reduced by Txnrd1 to Cys that protects cells from lipid peroxidation by removing ROS. Intracellular GSH is synthesized from its precursor amino acids catalyzed by glutamate–cysteine ligase (GCL) and GSH synthetase (GS). GSH protects cells from oxidative damage by removing ROS, and the resulting GSSG can be reduced to GSH by GSH reductase (GR). Both GSH and GSSG can be exported out of cells by the multidrug resistance protein (Mrp) transporter.

All aerobic organisms are subject to a certain level of oxidative stress due to the generation of cytotoxic reactive oxygen species (ROS) from mitochondrial respiration.^1^,^2^ Glutathione (GSH), the most abundant nonprotein thiol in mammalian cells, plays crucial roles in sustaining intracellular redox homeostasis by removing ROS.^3^ Notably, cancer cells intrinsically have higher levels of ROS due to the increased aerobic glycolysis,^4^,^5^ which also leads to higher GSH levels in order to adapt to this change and protect against ROS-induced apoptosis. However, this elevated GSH level also increases the resistance of cancer cells to pro-oxidant therapies,^6^,^7^ defined as those stressing the redox balance towards a more oxidized state, such as radio- and chemotherapies. As such, targeting GSH metabolism to deplete intracellular GSH by inhibiting glutamate–cysteine (Glu–Cys) ligase (GCL), a rate-limiting enzyme for GSH biosynthesis, has emerged as a promising strategy to sensitize cancer cells to chemotherapy.^8^–^11^ While GSH has traditionally been seen as the major antioxidant, recent studies revealed that intracellular GSH deficiency can be rescued by an independent redox system, *i.e.*, the cystine/cysteine (Cys_2_/Cys) redox cycle, characterized by the increased uptake of extracellular Cys_2_ (oxidized form of Cys), intracellular Cys_2_ reduction to Cys, and augmented intra- and extracellular Cys levels (Scheme 1).^12^–^14^ This redox cycle is driven by Cys_2_–Glu antiporter system x_c_^–^, and modulated by thioredoxin reductase 1 (Txnrd1) as well as alanine, serine, and Cys transporter ASCT, permitting cell survival and proliferation even when depleted of endogenous GSH. This implies that many GSH-related functions, such as ROS scavenging, protein modification, and cell signalling, can still occur as long as Cys supply is ensured. Also, this implies that only targeting GSH metabolism to deplete intracellular GSH is insufficient to sensitize cancer cells to chemotherapy, given that system x_c_^–^ is overexpressed in many types of tumors,^14^,^15^ which facilitates cancer cells to access to Cys *via* more abundant extracellular Cys_2_. In fact, blocking Cys_2_ uptake by inhibiting system x_c_^–^ has been shown to significantly increase the sensitivity of cancer cells to chemotherapies,^16^–^18^ even to be able to overcome drug resistance by inducing ferroptotic cell death.^19^–^21^ Overall, these studies indicate that targeting Cys metabolism, such as inhibiting the activity of system x_c_^–^ to deplete intracellular Cys, appears to be a more promising strategy for sensitizing tumors to chemotherapy, because Cys is not only the rate-limiting substrate for GSH biosynthesis, but also the key component of the Cys_2_/Cys redox cycle. In this context, the development of simple and reliable methods enabling sensitive, noninvasive, and real-time monitoring of intracellular Cys fluctuation is of great significance to evaluate relevant sensitizing agents for chemotherapy.

Among various cellular biology tools, fluorescent probes have shown unique advantages for mapping the spatial and temporal distributions of biomolecules due to their sensitivity, visualization, noninvasiveness, and real-time detection. To date, considerable efforts have been invested in the development of fluorescent Cys probes by exploiting Cys-triggered specific reactions,^22^,^23^ typically including cyclization with aldehydes,^24^–^26^ Michael addition–cyclization with acrylates,^27^–^29^ and S_N_Ar substitution–rearrangement.^30^–^43^ With these excellent strategies, the selective detection of Cys and even the simultaneous detection of Cys and GSH have been successfully realized [note that the interference caused by homocysteine (Hcy) is commonly negligible due to its significantly lower intracellular concentration (∼10 μM) than that of Cys (∼200 μM) or GSH (1–10 mM)]. However, as far as detecting Cys alone is concerned, some challenges still remain. For example, fluorescent Cys probes based on cyclization with aldehydes often suffer from poor reactivity, the need for organic solvents, and a long response time; and those based on Michael addition–cyclization and S_N_Ar substitution–rearrangement, although showing a fast and specific fluorescence response for Cys, may suffer from GSH-induced probe consumption, resulting in decreased sensitivity for Cys and adding uncertainty for assessing Cys-related physiological and pathological functions. Thus, it is necessary to develop more specific fluorescent Cys probes to overcome these limitations, especially the probe consumption caused by GSH. However, as far as we know, only a few fluorescent Cys probes hold such potential by exploiting either addition reaction combined with steric and electrostatic interactions or condensation reaction with a more specific thiobenzoate group.^44^–^47^

**Scheme 2:**
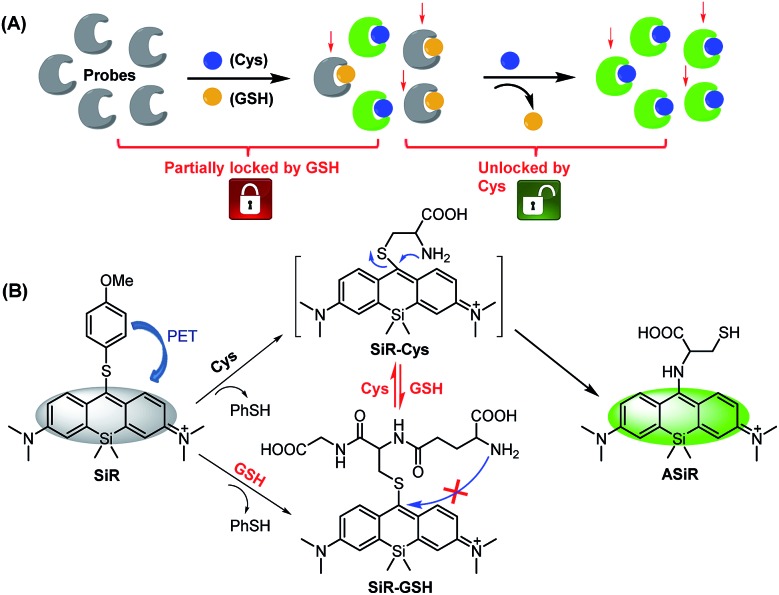
Design principle (A) and proposed sensing mechanism (B) of fluorescent probe **SiR** for Cys (PhSH = 4-methoxythiophenol).

In this work, we present a novel “lock and key” strategy to construct a highly selective fluorescent Cys probe for overcoming the above-mentioned limitations. As illustrated in Scheme 2A, the proposed probe would initially non-selectively react with both Cys and GSH, but resulting in the fluorescent Cys adduct and nonfluorescent GSH adduct, respectively. If the reactions stop at this stage, the probe consumption (*partially locked by GSH*), would be inevitable. However, if the nonfluorescent GSH adduct could further be displaced by Cys (*key*) to produce the fluorescent Cys adduct (*unlocked by Cys*), the GSH-caused probe consumption problem would be resolved. That is to say, the “lock and key” strategy could allow Cys to be detected without any signal interference and probe consumption caused by coexisting GSH. Based on this idea, we designed and synthesized a 4-methoxythiophenol-substituted Si-rhodamine **SiR** as a proof-of-concept application of the strategy (Scheme 2B). As expected, Cys could not only rapidly react with **SiR** to produce fluorescent amino-Si-rhodamine **ASiR***via* the well-established S_N_Ar substitution–rearrangement cascade,^30^–^43^ but also displace the GSH unit of the nonfluorescent adduct **SiR-GSH** to produce the same **ASiR**. As such, the presence of GSH would not cause any problem for specific detection of Cys. Importantly, with the probe, we not only demonstrated that inhibiting system x_c_^–^ is more efficient than inhibiting GCL for sensitizing cancer cells to chemotherapy, but also revealed a possible self-protection mechanism of cancer cells: when extracellular Cys sources are blocked, cancer cells can still survive by exporting intracellular GSH/GSSG as Cys sources to supply intracellular Cys for resisting detrimental oxidative stress. Finally, we confirmed that abrogating the self-protection is an even more efficient strategy for sensitizing cancer cells to chemotherapy.

## Results and discussion

### Spectral response of **SiR** for Cys

**Fig. 1:**
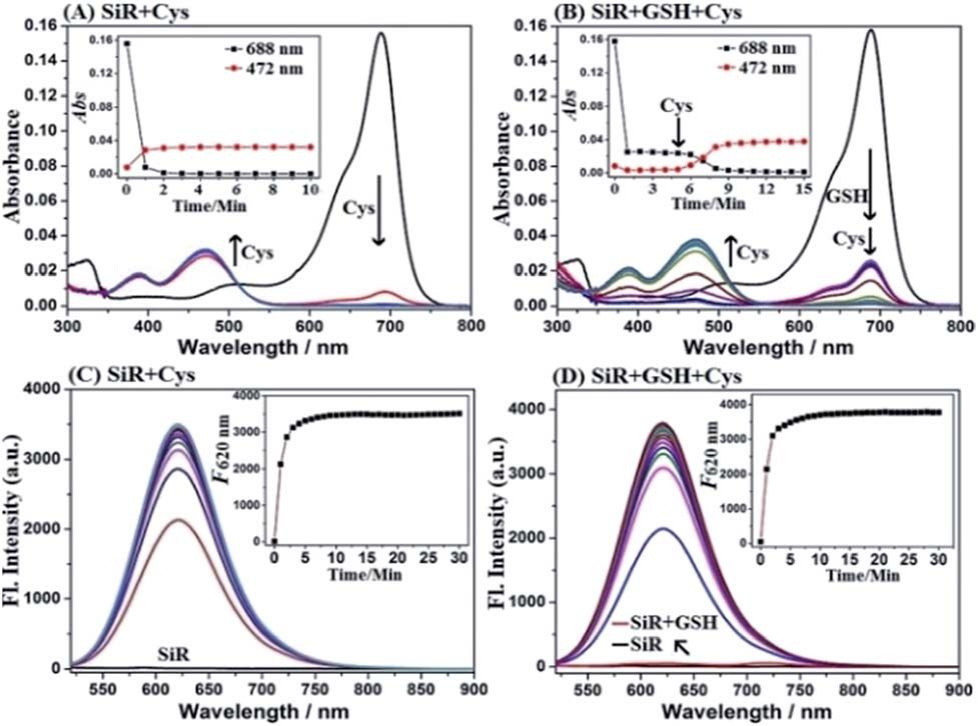
(A and B) Absorption spectra of **SiR** (2 μM) treated with Cys (20 μM) or pretreated with GSH (1 mM) for 5 min and then treated with Cys (20 μM) for 10 min. (C and D) Time-dependent fluorescence spectra of **SiR** (2 μM) treated with Cys (20 μM) for 30 min or pretreated with GSH (1 mM) for 5 min and then treated with Cys (20 μM) for 30 min. The spectrum was recorded every minute. Conditions: PBS (10 mM, pH 7.4); *λ*_ex_ = 488 nm and *λ*_em_ = 620 nm.

**SiR** was synthesized by a four-step procedure starting from commercially available materials (ESI†). With **SiR** in hand, we first tested its reactivity toward Cys and GSH in PBS (10 mM, pH = 7.4) using absorption spectra. As shown in Fig. 1A, upon the treatment with Cys, the initial absorption peak of **SiR** at 688 nm disappeared within 2 min, accompanied by the appearance of a new blue-shifted peak at 472 nm, which was assigned to amino-Si-rhodamine **ASiR** in terms of the well-established S_N_Ar substitution–rearrangement mechanism. In comparison, the GSH treatment only resulted in an obvious decrease of the initial absorption peak at 688 nm within 1 min (Fig. 1B), indicating that the reaction only produced thio-Si-rhodamine **SiR-GSH***via* initial S_N_Ar substitution and the subsequent macrocyclic S to N switching was unlikely.^22^,^31^ Importantly, upon the addition of Cys to the mixture of **SiR** and GSH, the absorption peak at 688 nm further dropped to baseline within 4 min, accompanied by the simultaneous appearance of a new blue-shifted peak at 472 nm, indicating that Cys could rapidly displace the GSH unit of **SiR-GSH** to produce **ASiR**. To confirm the above speculation, we analyzed the reaction products of **SiR** with Cys and GSH by HPLC-MS. As shown in Fig. S1 (ESI†), Cys (or GSH) treatment converted **SiR** to a new product, which could be assigned to **ASiR** (or **SiR-GSH**) based on MS data. However, upon further addition of Cys to the mixture of **SiR** and GSH, the initial HPLC peak of **SiR-GSH** disappeared and a new peak appeared, which was assigned to **ASiR** based on the retention time and MS. Obviously, the HPLC-MS results match well with the above absorption spectra studies, both supporting our proposed “lock and key” strategy.

Notably, amino-Si-rhodamines have recently been reported by us to be a new class of lysosome-targetable and red-emission fluorescent dyes with high quantum yields and large Stokes shifts.^48^ This, coupled with the above spectroscopic studies, implies that **SiR** should be an ideal fluorescent Cys probe that can avoid the issue of probe consumption caused by GSH. To confirm the speculation, we tested fluorescence spectra changes of **SiR** toward Cys and GSH. As shown in Fig. 1C, **SiR** itself was almost nonfluorescent when excited at 488 nm (*Φ* = 0.007). Upon the treatment with Cys, a new fluorescence peak appeared at 620 nm and reached a maximum within 5 min, consistent with the production of amino-Si-rhodamine **ASiR** (*Φ* = 0.13).^48^ By contrast, GSH treatment elicited almost no fluorescence enhancement under the same excitation, indicating that GSH would not cause fluorescence signal interference for Cys detection (Fig. 1D). Importantly, when Cys was subsequently added to the mixture of **SiR** and GSH, a dramatic fluorescence enhancement was also observed at 620 nm with almost the same reaction kinetics as that treated with Cys only, indicating that the probe consumption caused by the initial reaction of **SiR** with GSH would not impact Cys detection as well. These results were consistent with our proposed “lock and key” strategy. Furthermore, the fluorescence titration of **SiR** with Cys in the presence of 1 mM GSH revealed a good linear relationship between the fluorescence intensities at 620 nm and Cys concentrations in the range of 0–2 μM (Fig. S2, ESI†), and the detection limit for Cys was determined to be as low as 2.6 nM based on S/N = 3. Thus, in the presence of a high concentration of GSH, **SiR** would still show high sensitivity for Cys. The sensing performances of **SiR** for Cys in the presence of various biologically relevant species, including various amino acids, anions, and cations, were also tested. In fact, these species elicited either no fluorescence enhancement or no obvious interference for Cys detection (Fig. S3A, ESI†). Moreover, **SiR** also showed no any response to various ROS/RNS (Fig. S3B, ESI†). Further, **SiR** displayed negligible fluorescence in the pH range of 2–12, but had obvious fluorescence responses for Cys in the pH range of 6–8, thus being suitable for imaging applications at physiological pH (Fig. S4, ESI†). It should be noted that, due to the similar structure and reactivity of Hcy and Cys, **SiR** also exhibited similar absorption and fluorescence spectra changes toward Hcy (Fig. S5, ESI†). Even so, the interference caused by Hcy is negligible due to its very low even undetectable intracellular concentration [see Fig. S10, (ESI†) for quantification of intracellular biothiols]. Overall, these results indicate that **SiR** is a reliable fluorescent probe for real-time imaging of Cys fluctuation in complex biological environments.

To determine whether the “lock and key” strategy is also applicable to other fluorescent Cys probes of this kind, we tested the reactivity of two previously reported probes, *i.e.* NBD-Cl and Cy7-Cl (IR-780),^49^ toward Cys and GSH, as well as the reactivity of their GSH adducts toward Cys. As shown in Fig. S6 and S7 (ESI†), consistent with the reported results, the two probes could react with Cys and GSH to produce amino-substituted Cys adducts and sulfur-substituted GSH adducts, respectively. Notably, the GSH adduct, produced by the reaction of NBD-Cl and GSH, could further react with Cys to give rise to the Cys adduct within 30 min, whereas that produced by the reaction of Cy7-Cl and GSH only led to very small amounts of Cys adduct within 60 min when treated with Cys, indicating that the reaction site at the 4-position of NBD dye is more electrophilic than that at the *meso*-position of Cy7 dye to promote the replacement reaction. By comparison, the GSH adduct, produced by the reaction of our probe **SiR** with GSH, is more reactive toward Cys as indicated by the short reaction time of 5 min (Fig. 1B and D), consistent with the report that the *meso*-position of Si-rhodamine dye is electrophilic and susceptible to nucleophilic attack.^50^,^51^ Thus, the reactivity of the GSH adduct with Cys should strongly depend on the electrophilicity of reaction sites. Also, it is worth noting that the reaction of the GSH adduct with Cys indeed involves two steps, namely the initial reversible S_N_Ar substitution by Cys and subsequent irreversible S to N intramolecular rearrangement of the Cys unit (Scheme 2). Thus, the S to N intramolecular rearrangement of the Cys unit should also play a role in shifting reaction equilibrium to the right due to its irreversibility. Overall, these results indicate that, although not mentioned in previous reports, the reaction of the GSH adduct with Cys is possible to occur for other fluorescent probes of this kind in terms of the activity of their reaction sites.

### Imaging the fluctuation of intracellular Cys and comparing the sensitizing effect of SAS and BSO to chemotherapy

**Fig. 2:**
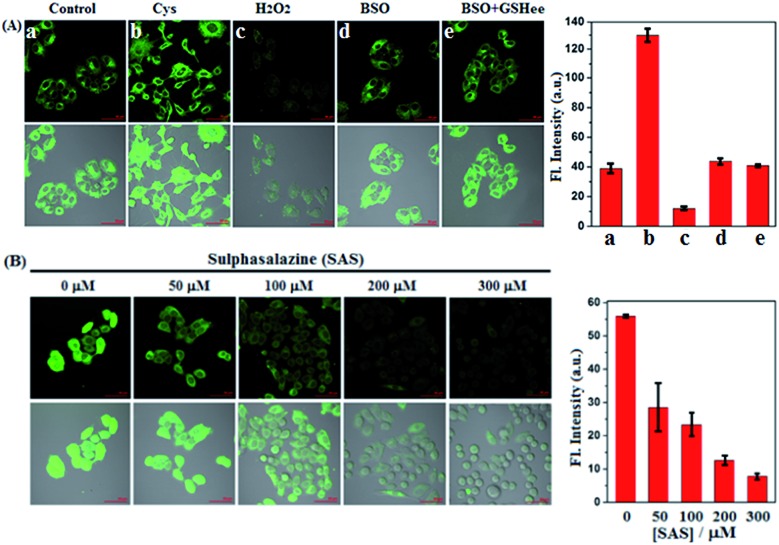
(A) Fluorescence images of HeLa cells pretreated with no agent, Cys (0.3 mM, 30 min), H_2_O_2_ (0.3 mM, 30 min), BSO (20 μM, 24 h), BSO (20 μM, 24 h)/GSHee (3 mM, 30 min), respectively, and then treated with **SiR** (4 μM, 15 min) in PBS. (B) Fluorescence images of HeLa cells pretreated with SAS (0–300 μM, 48 h) in DMEM and then treated with **SiR** (4 μM, 15 min) in PBS. Results are statistical analyses of >6 cells. Error bars represent standard deviations. Emission was collected at 550–650 nm (*λ*_ex_ = 488 nm). Scale bar: 50 μm.

Prior to biological imaging in living cells, the cytotoxicity of **SiR** was evaluated in human cervix carcinoma HeLa cells using a standard cell counting kit-8 (CCK-8) assay. As shown in Fig. S8 (ESI†), when incubated with various concentrations of **SiR** (0–20 μM) for 24 h, the cells showed high survival rates (more than 88%), confirming that **SiR** was almost nontoxic to living cells. Next, we tested the imaging performances of **SiR** for Cys in living HeLa cells. As shown in Fig. 2A(a–c), when HeLa cells were treated with **SiR**, a bright intracellular fluorescence was observed, indicating that **SiR** could penetrate the cell membrane and react with intracellular Cys to produce **ASiR**; by comparison, when HeLa cells were pretreated with exogenous Cys and then treated with **SiR**, the intracellular fluorescence became stronger; when HeLa cells were pretreated with H_2_O_2_, an oxidizing agent for biothiols, and then treated with **SiR**, the intracellular fluorescence was greatly decreased. The results indicate that **SiR** could be utilized to image the fluctuation of intracellular Cys. To test whether the intracellular abundant GSH would interfere with Cys detection using **SiR**, we performed imaging assays in HeLa cells pretreated with l-buthionine-sulfoximine (BSO), an inhibitor of GCL, which could deplete intracellular GSH but would not decrease the intracellular Cys level^12^,^52^ and has been evaluated in the phase I clinical trial.^53^ As shown in Fig. 2A(d and e), when HeLa cells were pretreated with BSO for 24 h to deplete intracellular GSH^12^ and then treated with **SiR**, a comparable intracellular fluorescence to that without BSO treatment was observed; moreover, when HeLa cells pretreated with BSO for 24 h to deplete intracellular GSH were further treated with 3 mM GSH ethyl ester (GSHee, a cell-permeant form of GSH that could increase intracellular GSH content)^54^,^55^ and then treated with **SiR**, we still observed a comparable intracellular fluorescence to that without GSHee treatment. Obviously, these results indicated that intracellular GSH did not affect the detection sensitivity of **SiR** for Cys. Further, we tested the ability of **SiR** in imaging intracellular Cys fluctuation induced by sulphasalazine (SAS), an inhibitor of system x_c_^–^,^56^–^58^ which has also been evaluated in the phase I clinical trial.^59^ As shown in Fig. 2B, when HeLa cells were pretreated with increasing concentrations of SAS for 48 h and then treated with **SiR**, a gradually decreased intracellular fluorescence was observed in a dose-dependent manner, consistent with previous reports that SAS could deplete intracellular Cys by inhibiting the activity of system x_c_^–^.^56^–^58^

**Fig. 3:**
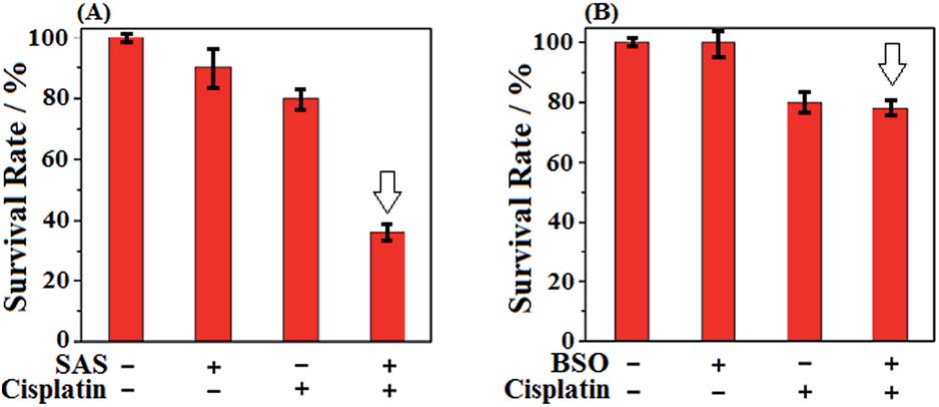
(A) Survival rates of HeLa cells treated with SAS (200 μM, 24 h) and cisplatin (1 μM, 24 h) in DMEM. (B) Survival rates of HeLa cells treated with BSO (20 μM, 48 h) and cisplatin (1 μM, 24 h) in DMEM. Results are from 6 replicates under the same conditions. Error bars represent standard deviations.

Given that SAS and BSO both were reported to be able to sensitize cancer cells to chemotherapies, we subsequently wanted to know which is better. Toward this end, we tested the sensitizing effect of SAS to chemotherapy in HeLa cells by CCK8 assays and compared the result with that of BSO. The classical anticancer drug cisplatin, which was known to produce high levels of ROS,^60^ was employed in the assays. As shown in Fig. 3A, when HeLa cells were treated with SAS (200 μM, a concentration that depleted intracellular Cys)^16^ for 24 h and then with cisplatin for 24 h, a dramatic decrease in cell viability (36%) relative to that observed in HeLa cells treated with cisplatin alone (80%) was found, indicative of the excellent sensitizing ability of SAS to chemotherapy. By comparison, as shown in Fig. 3B, when HeLa cells were treated with BSO (20 μM, a concentration that depleted intracellular GSH)^12^ for 24 h and then with cisplatin for 24 h, only a slightly decreased cell viability (78%) relative to that observed in HeLa cells treated with cisplatin (80%) alone was found, indicating that depleting intracellular GSH was not as efficient as depleting intracellular Cys in sensitizing cancer cells to chemotherapy. These results, together with the fact that Cys is not only the rate-limiting substrate for GSH biosynthesis but also the key component of the Cys_2_/Cys redox cycle, indicate that targeting Cys metabolism to deplete intracellular Cys should be a more efficient strategy in designing sensitizing agents for chemotherapy. Meanwhile, these results also indicate that **SiR** has the potential to evaluate relevant sensitizing agents for chemotherapies.

### Indicating a self-protection mechanism of cancer cells

**Fig. 4:**
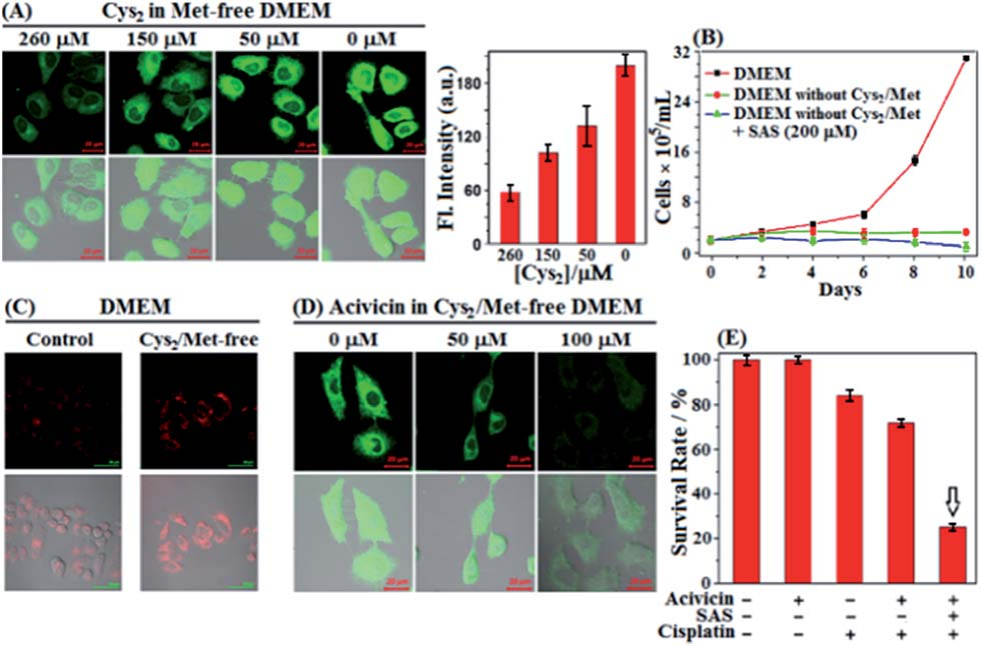
(A) Fluorescence images of HeLa cells cultured in Met-free DMEM containing decreased Cys_2_ contents (260–0 μM) for 2 days and then treated with **SiR** (4 μM, 15 min) in PBS. Results are statistical analyses of >10 cells, and error bars represent standard deviations. (B) Proliferation of HeLa cells in standard DMEM and in Met- and Cys_2_-free DMEM in the absence and presence of SAS (200 μM) during 10 days, respectively. Results represent mean values of three independent experiments with duplicate measurements ± s.d. (C) Immunofluorescence images of HeLa cells cultured in standard DMEM and in Met- and Cys_2_-free DMEM for 2 days, respectively, and then treated with Alexa Fluor 488 anti-human Mrp1 antibody. (D) Fluorescence images of HeLa cells treated with increased concentrations of acivicin in Met- and Cys_2_-free DMEM for 2 days, and then treated with **SiR** (4 μM, 15 min) in PBS. Average fluorescence intensities in (C and D) are shown in Fig. S12 (ESI†). For **SiR**, emission was collected at 550–650 nm (*λ*_ex_ = 488 nm); for the anti-human Mrp1 antibody, emission was at 500–700 nm (*λ*_ex_ = 488 nm). Scale bar: 20 μm. (E) Survival rates of HeLa cells treated with acivicin (5 μM, 48 h), SAS (200 μM, 48 h), and cisplatin (1 μM, 24 h), respectively, or their combination in DMEM. The assay results are from 6 replicates under the same conditions. Error bars represent standard deviations.

In next studies, we wanted to know whether cancer cells could still survive when extracellular Cys sources, including Cys/Cys_2_ and GSH/GSSG, were completely blocked [note that GSH/GSSG could be cleaved extracellularly to Cys/Cys_2_ by ectoenzymes GGT and DPs (Scheme 1)].^61^ To this end, we first performed imaging assays using **SiR** in HeLa cells cultured in a Met-free DMEM medium containing a decreased Cys_2_ content for 48 h. Of note, the standard DMEM medium contained Cys_2_ (∼260 μM) but no other Cys sources, such as Cys/GSH/GSSG, and the intentional removal of Met in DMEM medium was to block the possible Cys supply *via* the *trans*-sulfuration pathway that mainly occurred in liver cells.^6^ To our surprise, as the extracellular Cys_2_ contents decreased from standard 260 μM to 0 μM, the fluorescence intensities of HeLa cells gradually increased and reached a maximum when the extracellular Cys_2_ content dropped to 0 μM (Fig. 4A). A similar case was also observed when the human A549 non-small-cell lung cancer cell line was employed (Fig. S9, ESI†). The results seem to indicate that blocking extracellular Cys sources could induce an increase of intracellular Cys levels. To support the speculation, we measured the changes of intracellular Cys levels in the lysates of HeLa cells incubated in standard and Met/Cys_2_-free DMEM medium for 48 h, respectively, using the enzyme-linked immune response (ELISA) kit. The obtained results showed that the intracellular Cys level increased up to 2.35 fold when extracellular Cys sources were completely blocked (Fig. S10, ESI†). In addition, using commercial fluorescent ROS probe 2′,7′-dichlorofluorescein diacetate (DCF-DA), we also found that when the extracellular Cys_2_ was blocked, the intracellular ROS level gradually increased and reached a maximum at 24 h, and then decreased after 48 h (Fig. S11, ESI†), indicating that the increase of the intracellular Cys level is probably to adapt to this change and protect against ROS-induced apoptosis. Importantly, it was found that when extracellular Cys_2_ was completely blocked, HeLa cells could still survive for at least 10 days, although they did not proliferate obviously when compared to those cultured in normal DMEM (Fig. 4B). These findings appear to indicate that in the absence of extracellular Cys_2_, HeLa cells started a self-protection mechanism to resist detrimental oxidative stress by increasing the intracellular Cys level. But what causes the elevation of the intracellular Cys level? How does the self-protection mechanism work? Notably, when incubated in Met/Cys_2_-free DMEM medium for 10 days in the presence of system x_c_^–^ inhibitor SAS, most of the HeLa cells were found to die (Fig. 4B), indicating that the self-protection mechanism could be related to the function of system x_c_^–^, *i.e.* uptake of extracellular Cys_2_ to supply intracellular Cys. However, the fact is that the conditioned DMEM medium does not contain any Cys_2_. So where does the extracellular Cys_2_ come from?

We noticed that cells undergoing oxidative stress release GSH/GSSG into the extracellular space.^62^–^64^ Thus, we reasoned that the intracellular GSH/GSSG export should be responsible for the above-mentioned production of extracellular Cys_2_ and the elevation of the intracellular Cys level (Scheme 1). In fact, using the enzyme-linked immune response (ELISA) kit, we observed an obviously decreased intracellular GSH level of HeLa cells when incubated in Met/Cys_2_-free DMEM medium for 2 days (Fig. S10, ESI†); moreover, using immunofluorescence staining and western blotting, we also observed an obviously up-regulated multidrug resistance protein transporter Mrp1, responsible for intracellular GSH/GSSG export,^6^,^65^ in HeLa cells cultured in Met- and Cys_2_-free DMEM for 2 days (Fig. 4C and S13 (ESI†)). These results indicate that when extracellular Cys_2_ was completely blocked, HeLa cells up-regulated the expression of Mrp1 to promote the export of intracellular GSH/GSSG. Further, we tested the relationship between the intracellular Cys levels and the activity of GGT, an ectoenzyme that, coupled with DPs, cleaves extracellular GSH/GSSG to Cys/Cys_2_ (Scheme 1).^58^ It was found that when HeLa cells were pretreated with acivicin, an inhibitor of GGT,^66^ in Met- and Cys_2_-free DMEM for 2 days and then treated with **SiR**, the intracellular fluorescence dramatically decreased relative to that observed in HeLa cells without acivicin treatment (Fig. 4D), strongly indicating that inhibiting the activity of GGT could prevent HeLa cells from utilizing the exported GSH/GSSG as sources to supply intracellular Cys. Based on these observations, we proposed the self-protection mechanism of HeLa cells in the absence of extracellular Cys sources: the export of intracellular GSH/GSSG *via* Mrp1, conversion of the exported GSH/GSSG to Cys/Cys_2_*via* GGT and DPs, uptake of the produced Cys/Cys_2_ by the ASCT transporter and system x_c_^–^, and removal of ROS by the Cys_2_/Cys redox cycle (Scheme 1). Further, we speculated that abrogating the self-protection mechanism should be able to sensitize cancer cells to chemotherapy more efficiently. As expected, when treated with acivicin and SAS to simultaneously inhibit the activity of GGT and system x_c_^–^ and then treated with cisplatin in standard DMEM, HeLa cells showed an obviously decreased survival rate (25%) relative to those treated with either acivicin/cisplatin (72%) (Fig. 4E) or SAS/cisplatin (36%) (Fig. 3A).

## Conclusions

In summary, in this work a “lock and key” strategy was presented to construct highly selective and sensitive fluorescent Cys probes. The resulting probe, *e.g.***SiR**, was able to detect Cys without any signal interference and probe consumption caused by the high concentration of intracellular GSH. With this probe we studied the regulatory roles of Cys in cancer cell survival. It was found that depleting intracellular Cys was more efficient than depleting intracellular GSH in sensitizing cancer cells to chemotherapies. Moreover, using the probe, a possible self-protection mechanism of cancer cells was proposed, that is, when extracellular Cys sources were completely blocked, cancer cells could still survive by exporting intracellular GSH/GSSG as sources to supply intracellular Cys for resisting detrimental oxidative stress. Further, we confirmed that abrogating the self-protection by simultaneous inhibition of the activity of GGT and system x_c_^–^ is an even more efficient strategy for sensitizing cancer cells to chemotherapy. Given that human blood plasma contains not only Cys_2_ but also GSH/GSSG, we envision that the combinational treatment should also be efficient for *in situ* sensitizing tumors to chemotherapy. Even so, there is still a long way to go, because most of the current inhibitors of GGT and system x_c_^–^ are more or less toxic for clinical uses.^67^,^68^ In this sense, our probe should be useful for screening and evaluating relevant sensitizing agents for chemotherapies in future.

## Conflicts of interest

There are no conflicts to declare.

## Supplementary Material

Supplementary information
