# Supplementary material for: Exploring cysteine regulation in cancer cell survival with a highly specific “Lock and Key” fluorescent probe for cysteine
Source: Chem Sci. 2019 Sep 7;10(43):10065–71. doi: 10.1039/c9sc02618e (PMC6991186; doi:10.1039/c9sc02618e)
Supplement: Supplementary file 1 [file SC-010-C9SC02618E-s001.pdf]

Electronic Supplementary Material (ESI) for Chemical Science.
This journal is © The Royal Society of Chemistry 2019

Supporting Information

Exploring Cysteine Regulation in Cancel Cell Survival with a Highly Specific "Lock and Key" Fluorescent Probe for Cysteine

Jing Liu,[a] Mengxing Liu,[a] Hongxing Zhang,[a] Xuehong Wei,[b] Juanjuan Wang,[b] Ming Xian,[c,]* and Wei Guo[a,]*

[a] *School of Chemistry and Chemical Engineering, Shanxi University, Taiyuan 030006, China.*

[b] *Scientific Instrument Center, Shanxi University, Taiyuan 030006, China.*

[d] *Department of Chemistry, Washington State University, Pullman, WA 99164 (USA)*

Corresponding Author E-mail: guow@sxu.edu.cn

1. General methods

All reagents and solvents were purchased from commercial sources and were of the highest grade. LysoTrackerDeep Red, MitoTrackerDeep Red FM, Dulbecco's Modified Eagle's Medium (DMEM, High glucose), Dulbecco's Modified Eagle's Medium (DMEM, High glucose, no Cystine and Methionine) were purchased from Invitrogen (USA). Sulfasalazine (SAS), Acivicin, L-buthionine-sulfoximine (BSO), and cis-Diammineplatinum (II) (cisplatin) were purchased from Innochem (China). Alexa Fluor 488 anti–human Mrp1 (ABCC1) was purchased from Biolegend (USA). Human Cys/Hcy/GSH ELISA Kits were purchased from Shanghai Enzyme-linked Biotechnology Co., Led. (China). Reactive Oxygen Species Assay Kit (DCFH-DA) was purchased from Solarbio. Rabbit Anti-MRP1/ABCC1 Polyclonal Antibody, Rabbit anti-β-Actin Polyclonal Antibody and HRP-labeled Goat Anti-Rabbit IgG were purchased from Bioss. NBD-Cl was purchased from Innochem (China). All cell lines were purchased from GeneFull Biotech co., Ltd (China). Solvents were dried according to standard procedures. All reactions were magnetically stirred and monitored by thin–layer chromatography (TLC). Flash chromatography was performed using silica gel 60 (200–300 mesh). Absorption spectra were taken on a

Varian Carry 4000 spectrophotometer. Fluorescence spectra were taken on Hitachi F–7000 fluorescence spectrometer. The ^{1}H NMR and ^{13}C NMR spectra were recorded at 600 and 150 MHz, respectively. The following abbreviations were used to explain the multiplicities: s = singlet; d = doublet; t = triplet; q = quartet; m = multiplet; br = broad. High resolution mass spectra were obtained on a Varian QFT–ESI mass spectrometer. For living cell fluorescence imaging assays, the images were acquired by Ceiss LMS 880 + Airyscan confocal microscope.

2. Synthesis

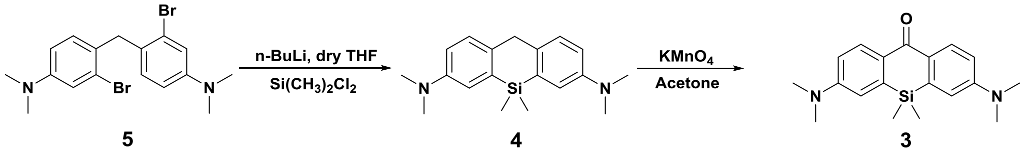

Compound 3: 4,4'-Methylenebis(3-bromo-*N*,*N*-dimethylaniline) **5** (6.00 g, 14.6mmol) was dissolved in dry THF (200 ml) and stirred at –78 °C. *n*-BuLi (2.4 M in *n*-hexane, 24.3 ml, 75.36 mmol, 4 equiv) was slowly added for 30 min to the solution and stirred for further 2 h at the same temperature. $SiMe_2Cl_2$ (3.2 ml, 26.28 mmol, 1.8 equiv) was added to the reaction mixture and then stirred at room temperature for 2 h. 1N HCl aqueous solution was added carefully to neutralize the solution, and then THF was evaporated. The resulting aqueous solution was extracted with EtOAc, and the organic phase was washed with saturated $NaHCO_3$ aqueous solution, water and brine, dried over Na_2SO_4, filtered and evaporated to obtain crude **4**. Crude **4** was dissolved in acetone (30 ml) and stirred at -15 ℃. $KMnO_4$ powder (5.75 g) was added portionwise for 30 min, and stirring was continued for further 2 h at the same temperature. The purple suspension was filtered through a Celite pad, and the yellow filtrate was evaporated. The residue was purified by silica gel column chromatography (CH_2Cl_2) to give **3** as a yellow solid (1.65 g, 34.7%). ^{1}H NMR (600 Hz, $CDCl_3$) δ 8.44 (d, *J* = 9.0 Hz, 2H), 6.87 (d, *J* = 9.0 Hz, 2H), 6.83 (s, 2H), 3.11 (s, 12H), 0.49 (s, 6H); ^{13}C NMR (150 MHz, $CDCl_3$) δ 185.3, 151.4, 140.5, 131.6, 129.7, 114.3, 113.2, 40.1, 0.97; ESI-MS $[M+H]^+$:Calcd for 325.1736, Found 325.1734.

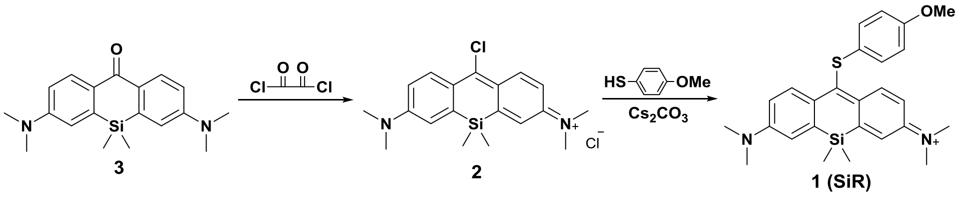

Compound 1 (SiR): Oxalyl chloride (1.2 eq.) was added to a solution of **3** (0.324 g, 1 mmol) in dry CH_2Cl_2 (10 mL). The reaction mixture was stirred for 10 min at r.t.. After the reaction was completed, the solution was evaporated. The crude product was purified by flash chromatography (CH_2Cl_2/CH_3CN = 5/2) to give the chloride species **2** (0.313 g, Yield 82.8%). ^{1}H NMR (600 Hz, $CDCl_3$) δ 8.43 (d, J = 9.6 Hz, 2H), 7.14 (s, 2H), 6.97 (d, J = 9.6 Hz, 2H), 3.48 (s, 12H), 0.58 (s, 6H). ^{13}C NMR (150 MHz, $CDCl_3$) δ 162.8, 154.4, 147.5, 139.7, 126.2, 120.2, 115.2, 41.5, 0.8; ESI-MS $[M]^+$: Calcd for 343.1392, Found 343.1391. The mixture of 4–methoxyphenol (52 mg, 45.6 μL, 0.37 mmol) and Cs_2CO_3 (121 mg, 0.37 mmol) in CH_2Cl_2 (10 mL) was stirred for 30 min, then **2** (0.189 g, 0.5 mmol) in CH_2Cl_2 (10 mL) was added. The reaction mixture was further stirred until the solution turned to colorless, and then Cs_2CO_3 was filtered. The filtrate was added dropwise to trifluoroacetic acid (TFA, 100 mL). The mixture was stirred for 10 min at 25 ^{o}C. The solvents were removed under reduced pressure and the residue was purified by flash chromatography (CH_2Cl_2/MeOH = 15/1) to afford pure product **1** (**SiR**) (147 mg, Yield 82.3%).^{1}H NMR (600 Hz, $CDCl_3$) δ 8.66 (d, J = 8.4 Hz, 2H), 7.22 (d, J = 8.4 Hz, 2H), 7.08 (d, J = 3.0 Hz, 2H), 6.84 (d, J = 8.4 Hz, 2H), 6.73 (dd, J_1= 2.4 Hz, J_2= 9.6 Hz,2H), 3.78 (s, 3H), 3.37 (s,12H), 0.54 (s, 8H); ^{13}C NMR (150 MHz, $CDCl_3$) δ 171.0, 159.3, 153.9, 147.2, 140.9, 131.5, 129.8, 128.2, 119.9, 115.5, 114.0, 55.4, 40.9, 1.4; ESI-MS $[M]^+$: Calcd for 447.1921, Found 447.1936.

Cy7-Cl (IR-780): Cy7-Cl was prepared according to the reported method[1] and its structure was confirmed by ^{1}H NMR. ^{1}H NMR (600 Hz, CDCl3) δ 8.36 (d, J = 13.8 Hz, 2H), 7.42 (m, 4H), 7.25 (t, J = 7.2 Hz, 2H), 7.21 (d, J = 8.4 Hz, 2H), 6.24 (d, J = 13.8 Hz, 2H), 3.76 (s, 6H), 2.76 (t, J = 6.6 Hz, 4H), 1.99 (m, 2H), 1.73 (s, 12H).

3. Preparation of the test solution

A stock solution of **SiR** (2 mM) was prepared in CH_3CN. The stock solution of **SiR** was then diluted to the corresponding concentration (2 μM) with PBS buffer (10 mM, pH 7.4). Fresh SAS solutions were prepared every day in 0.1 N NaOH and subsequently adjusted with 1 N HCl to a pH of about 8. BSO and Acivicin were dissolved in deionized water. Cis-Diammineplatinum (II) was dissolved in minimum–volume *N,N*-dimethylformamide (DMF), which was then diluted with physiological saline. The solutions of cysteine (Cys), homocysteine (Hcy), glutathione

(GSH) and various amino acids were prepared in deionized water. The aqueous solutions of anions were freshly prepared from their sodium salts in deionized water, and the aqueous solutions of cations were freshly prepared from their chloride salts in deionized water.

4. Quantum yield determination

Fluorescence quantum yields of **SiR** and **ASiR** (**SiR**+Cys) were determined in PBS, with fluorescein (Φ_f = 0.95 in 0.1 M NaOH) as standard. The quantum yield was calculated using Eq.1:

$\Phi_u = [(A_s FA_u \eta^2)/ (A_u FA_s \eta_0^2)]\Phi s.$ (Eq.1)

Where A_s and A_u are the absorbance of the reference and sample solution at the reference excitation wavelength, FA_s and FA_u are the corresponding integrated fluorescence intensity, and η and η_0 are the solvent refractive indexes of sample and reference, respectively. Absorbance of sample and reference at their respective excitation wavelengths was controlled to be lower than 0.05. The fluorescence quantum yields of **SiR** and **ASiR** (**SiR**+Cys) were determined to be 0.007 and 0.13, respectively.

5. Cytotoxicity assays of SiR

The CCK–8 cell proliferation assay was applied to investigate the cytotoxicity of **SiR**. Briefly, the growth adherent HeLa cells were digested into cell suspensions. Cells were seeded at a density of 5.0×10^3 cell per well into a 96–well plate and incubated with 100 μL of culture media overnight for cell attachment. Then, the stock solution of **SiR** (2 mM) in CH_3CN was added, and the final concentrations of **SiR** were kept from 0 to 20 μM for further incubation with 24 h. Six replicate wells were used for each control and test concentration. Subsequently, cells were washed twice with PBS and incubated with fresh medium containing 10 μL CCK–8 for 0.5 h. The absorbance at 450 nm was measured by *iMark^{TM}MicroplateAbsorbance Reader*. Cell viability (%) = ($A_{with\ probe}$−A_{blank} / $A_{control}$−A_{blank}) × 100%.

6. Subcellular localization

HeLa cells were grown in DMEM (High glucose) supplemented with 10 % FBS (Fetal Bovine Serum), 100 U/mLsodium penicillin G and 100 μg/mL streptomycin at 37 ℃ in humidified environment of 5% CO_2. Cells were plated on glass bottom cell culture dish (30 mm) and allowed to adhere for 12 hours. Before experiments, HeLa cells were washed with phosphate buffer saline (PBS) 3 times. Then, the cells were costained with **SiR** (2 μM, 15 min) and LysoTrackerDeep Red FM (0.5 μM, 10 min), or MitoTrackerDeep Red (0.5 μM, 10 min) in PBS at 37 ℃. After washed with PBS 3 times, fluorescence images were performed. Emission was collected at 530–630 nm for **SiR** (excited at 488 nm), and at 650–750 nm for LysoTrackerDeep Red FM and MitoTrackerDeep Red (excited at 633 nm).

7. Imaging endogenous and exogenous Cys in HeLa cells

HeLa cells were grown in DMEM (High glucose) supplemented with 10 % FBS and and 1% antibiotics at 37 ℃ in humidified environment of 5% CO_2. Cells were plated on glass bottom cell culture dish (30 mm) and allowed to adhere for 12 hours. Before experiments, cells were washed with phosphate buffer saline (PBS) 3 times. In control assay, cells were only treated with **SiR** (4μM, 15 min) in PBS. To test whether **SiR** could image the fluctuation of Cys, HeLa cells were pretreated with Cys (0.3 mM, 30 min) or H_2O_2 (0.3 mM, 30 min) and then treated with **SiR** (4 μM, 15 min) in PBS. To test whether the intracellular abundant GSH interferes with the Cys detection, HeLa cells were pretreated with BSO (20 μM, 24 hr) in DMEM and then treated with or without GSHee (3 mM, 30 min) in PBS, followed by the treatment with 4 μM **SiR** in PBS for 15 min. After washed with PBS 3 times, these cells were imaged under Ceiss LMS 880+Airyscan confocal microscope with a 60×oil–immersion objective lens. Emission was collected at 550–650 nm (excited at 488 nm).

8. Imaging intracellular Cys fluctuation induced by SAS and comparing the sensibilization effect of SAS and BSO to pro-oxidant anticancer therapy

(1) HeLa cells were plated on glass bottom cell culture dish (30 mm) and grown in DMEM (High glucose) supplemented with 10 % FBS (Fetal Bovine Serum) and 1%

antibiotics at 37 ℃ in humidified environment of 5% CO_2 to adhere for 12 hours. To test the ability of **SiR** in imaging intracellular Cys fluctuation induced by SAS, HeLa cells were pretreated with the increased concentrations of SAS (0–300 μM) for 48 h in DMEM and then treated with **SiR** (4μM, 15 min) in PBS. After washed with PBS 3 times, the fluorescence imaging assays were performed. Emission was collected at 550–650 nm (excited at 488 nm).

(2) The CCK–8 cell proliferation assay was applied to determine the SAS/BSO induced sensitization of HeLa cells to cisplatin. The growth adherent cells were digested into cell suspensions. HeLa cells suspensions with a density of 5.0×10^3 cells per well in 100 μL cell culture medium were inoculated into 96–well plates. After cell attachment, the cells were treated with no drugs (control), SAS (200 μmol/L, 48 h) or BSO (20 μmol/L, 48 h), cisplatin (1μmol/L,24 h), respectively; or treated with BSO (20 μM) or SAS (200 μM) for 48 hr and at 24 hr treated with cisplatin (1 μM, 24 hr). Subsequently, cells were washed twice with PBS and incubated with fresh medium containing 10 μL CCK–8 for 0.5 h. Six replicate wells were used for each test. The absorbance at 450 nm was measured by *iMark*^{TM}*MicroplateAbsorbance Reader*. Cell viability (%) = $(A_{with\ probe}-A_{blank} / A_{control}-A_{blank}) \times 100\%$.

9. Revealing a self-protection mechanism of HeLa cells when extracellular Cys sources are completely blocked

(1) To test whether HeLa cells can survive when extracellular Cys sources, such as Cys, Cys_2, GSH, and Met, are completely blocked, the cells were plated on glass bottom cell culture dish (30 mm) and cultured in Met–free DMEM (High glucose) containing the decreased Cys_2 content (260 μM, 150 μM, 100 μM, 50 μM, 0 μM) supplemented with 10 % FBS and 1% antibiotics at 37 ℃ in humidified environment of 5% CO_2 for 2 days, followed by the treatment with **SiR** (4μM, 15 min) in PBS. Of note, the standard DMEM medium contains Cys_2 (~260 μM) but no Cys/GSH, and the intentional removing of Met in DMEM medium is to block the possible Cys supply via trans-sulfuration pathway that is indeed only active in liver cells. After washed with PBS 3 times, imaging assays were performed under Ceiss LMS 880 +

Airyscan confocal microscope. Emission was collected at 550–650 nm (excited at 488 nm).

(2) To assess the proliferation of HeLa cells in standard DMEM and in Met/Cys_2–free DMEM, 150,000 cells were seeded into each well of a 6–well plate, the number of viable cells was determined over a period of 10 days. Cells grown in DMEM or in Met/Cys_2–free DMEM were harvested using 0.05% trypsin and resuspended in 10 ml of serum–free DMEM. Three readings were made on specified days (0, 2, 4, 6, 8 and 10 days) using a blood count board.

(3) To confirm the speculation that the increase of intracellular Cys level in Met/Cys_2-free DMEM is due to the export of intracellular GSH/GSSG and their extracellular metabolism via γ-glutamyl cycle, the expression level of multidrug resistance protein transporter Mrp1, responsible for intracellular GSH/GSSG export in γ-glutamyl cycle, was determined in HeLa cells by the direct immunofluorescence using Alexa Fluor 488 anti–human Mrp1 (ABCC1) antibody. In this assay, HeLa cells were grown in standard DMEM (High glucose) and Met/Cys_2–free DMEM (High glucose) for 48 h, respectively. Then, the cells were treated with Alexa Fluor 488 anti–human Mrp1 (ABCC1) antibody (2 μL, PBS, pH 7.2, containing 0.09% sodium azide and 0.2% BSA) for 30 min at 4 ℃. After washing with PBS, 60× images were acquired using a confocal microscopy (Zeiss, LSM880+Airyscan). Images were collected at 500–700 nm (excited at 488 nm). Also, the expression of MRP1 was checked by Western blotting. In this assays, HeLa cells seeded in six-well plates (5×10^4 cells/well) were grown in DMEM or Cys_2/Met-free DMEM supplemented with 10 % FBS and 1% antibiotics at 37 ℃ in humidified environment of 5% CO_2 for 2 days. 10 μg of total cell lysate was resolved on Any kD Mini-PROTEAN TGX Stain-Free Gel (Bio-Rad) using the Laemmli gel system. Proteins separated by SDS-PAGE were then transferred to low fluorescence PVDF membrane and incubated with the primary antibody against MRP1 diluted 1:200 into 10 mL with Skimmed Milk Powder overnight, or anti-β-actin antibody (at 1:1500, v/v dilution). Low fluorescence PVDF membranes were washed with TBST, incubated with HRP-labeled Goat Anti-Rabbit IgG (secondary antibody, at 1:5000, v/v dilution) for 1 h and then

washed with TBST. ChemiDoc MP (Bio-Rad) was used to detect the chemiluminescent signal in Western blotting.

(4) To test the relationship between the intracellular Cys level and the activity of GGT (an ectoenzyme that, coupled with DPs, cleaves extracellular GSH/GSSG to Cys/Cys_2), HeLa cells were plated in a 30 mm diameter dish and allowed to adhere in Met/Cys_2–free DMEM supplemented with 10% FBS. After various concentrations of Acivicin (50–100 μM) were added to the culture, the cells were re–cultured for 2 days. The cells were washed twice with PBS and then treated with 4 μM **SiR** in PBS for 15 min. After washing with PBS, 60× images were latter acquired using a confocal microscopy (Zeiss, LSM880+Airyscan). Images were collected at 550–650 nm (excited at 488 nm).

(5) The CCK–8 cell proliferation assay was applied to determine the Acivicin or Acivicin/SAS induced sensitization of HeLa cells to cisplatin in standard DMEM. The growth adherent cells were digested into cell suspensions. HeLa cells suspensions with a density of 5.0×10^3 cells per well in 100 μL cell culture medium were inoculated into 96–well plates. After cell attachment, the cells were treated with no drugs (control), Acivicin (5 μmol/L, 48 hr), and cisplatin (1 μmol/L, 24 h), respectively; or treated with Acivicin (5 μmol/L) or Acivicin (5 μmol/L)/SAS (200 μmol/L) for 48 hr and at 24 hr treated with cisplatin (1 μM, 24 hr) in the standard DMEM. Subsequently, cells were washed twice with PBS and incubated with fresh medium containing 10 μL CCK–8 for 0.5 h. Six replicate wells were used for each test. The absorbance at 450 nm was measured by *iMark^{TM}MicroplateAbsorbance Reader*. Cell viability (%) = $(A_{with\ probe} - A_{blank} / A_{control} - A_{blank}) \times 100\%$.

10. Comparison of the Cys and GSH levels in the lysates of HeLa cells incubated in standard and in Met/Cys_2-free DMEM medium for 2 days, respectively

Human Cys/GSH ELISA Kid was used to measure the concentrations of Cys, Hcy, and GSH in the lysates of HeLa cells incubated in standard DMEM or in Cys_2/Met-free DMEM. The absorbance changes were obtained for a range of standard Cys, Hcy, and GSH concentrations to generate a standard curve of optical density

(450 nm) versus Cys, Hcy, and GSH concentrations. The concentrations of Cys, Hcy, and GSH in the samples were then determined by comparing the optical density of the samples to the standard curve. Specifically, HeLa cells were pre-incubated in standard DMEM or Met/Cys_2–free DMEM for 48 h, and then the cells were suspended with a density of 1.0×10^6 cells/mL and sonified in 1 mL PBS (10 mM, pH 7.4). After centrifugation 20 min at the speed of 2000–3000 r.p.m., the supernatant was collected and assayed for Cys, Hcy, and GSH using Human Cys, Hcy, and GSH ELISA Kit according to the procedures recommended by the manufacturer. Cys, Hcy, and GSH concentrations were calculated using the standard curve.

11. Determination of intracellular ROS

Cellular ROS were measured using the fluorogenic reagent 2',7'-dichlorodihydrofluorescein diacetate (DCFH-DA). Briefly, Cells were plated on glass bottom cell culture dish (30 mm) and grown in standard DMEM or Met/Cys_2–free DMEM supplemented with 10 % FBS and 1% antibiotics at 37 ℃ in humidified environment of 5% CO_2 for 2 days. ROS levels were determined by loading cells with 10 μM DCFH-DA for 30 min. Cells were washed twice with PBS and then imaged under Ceiss LMS 880+Airyscan confocal microscope with a 60×oil–immersion objective lens. Emission was collected at 500–600 nm (excited at 488 nm).

12. Supplementary Spectra and Imaging data

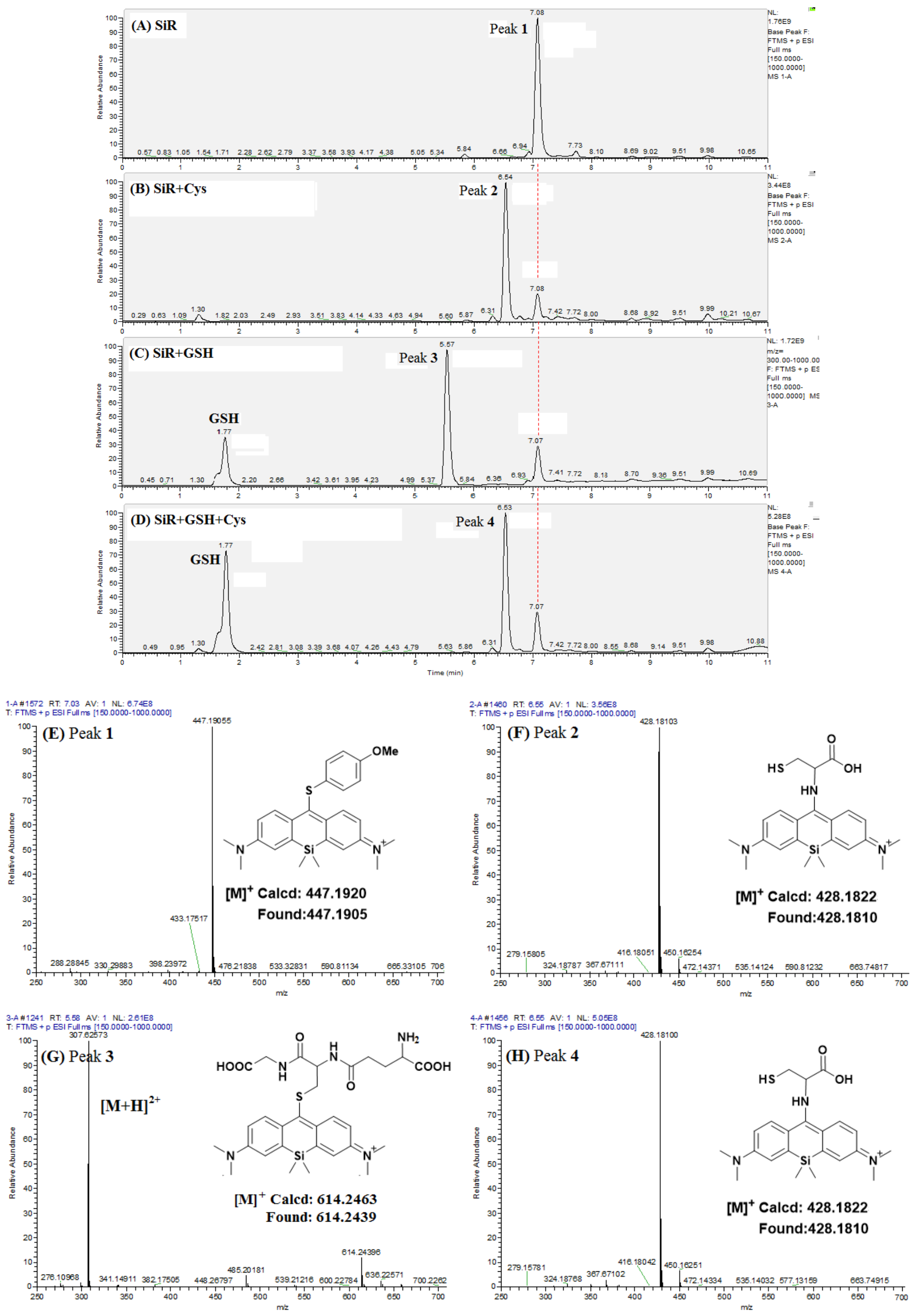

Figure S1. (A-D) HPLC charts of **SiR** (4 μM), **SiR** treated with 400μM of Cys, **SiR** treated with1 mM of GSH, **SiR** treated with 1 mM of GSH then treated with 400 μM of Cys in deionized water, respectively. HPLC condition: C18 column (2.1 × 100 mm); mobile phase: MeCN/H_2O (9:1 to 1:0); Flow rate: 0.3 mL/min. (E-H) HRMS charts of peaks 1-4 in (A-D).

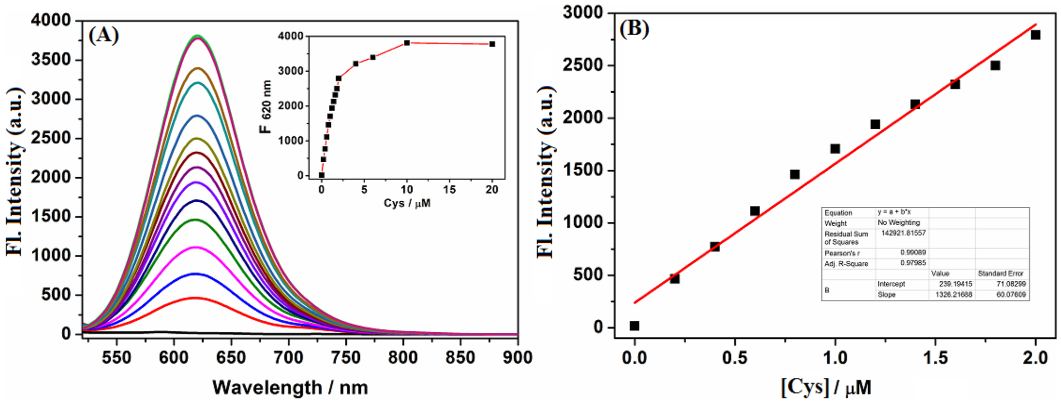

Figure S2.(A) Fluorescence spectra changes of **SiR** (2μM) pretreated with 1 mM GSH for 5 min and then treated with the increasing concentrations of Cys (0–20 μM). Each spectrum was recorded at the time point of 10 min after the addition of Cys. (B) Plot of the fluorescence intensities of **SiR**(2 μM) at 620 nm as a function of Cys concentrations (0–2 μM) .Conditions: PBS (10mM, pH 7.4) at 25 °C; λ_{ex} = 488 nm; λ_{em} = 620 nm; Slits: 5/10 nm; voltage: 700 V.

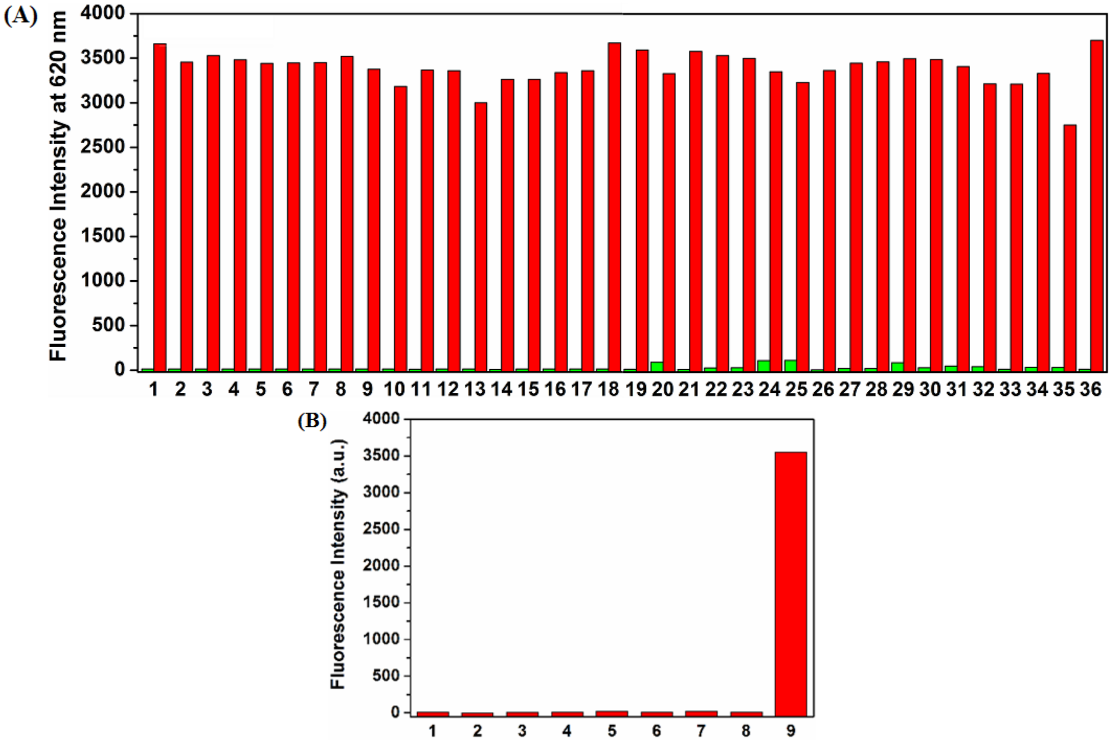

Figure S3. (A) Fluorescence intensities of **SiR** (2.0 μM) treated with Cys (10 equiv) in the absence and presence of 10 equiv of various species.(1) **SiR** only; (2) Na^+;(3) Cu^{2+}; (4) Hg^{2+}; (5) Pb^{2+}; (6) Ca^{2+}; (7) K^+; (8) Mg^{2+}; (9) Zn^{2+}; (10) SO_3^{2-}; (11) HCO_3^-;

(12) NO_3^-; (13) S^{2-}; (14) Cl^-; (15) Br^-; (16) I^-; (17) SO_4^{2-}; (18) His; (19) Glu; (20) Asp; (21) Val; (22) Phe; (23) Tyr; (24) Ala; (25) Ser; (26) Leu; (27) Arg; (28) Lys; (29) Pro; (30) Thr; (31) Asn; (32) L-Glu; (33) Trp; (34) Ile; (35) Lys; (36) GSH. Green bar: **SiR**+various species; red bar: **SiR**+various species+Cys. (B) Fluorescence intensities of **SiR** (2.0 μM) treated with 10 equiv of various ROS and Cys. (1) **SiR** only; (2) ClO^-; (3) $ONOO^-$; (4) H_2O_2; (5) $O_2^{\bullet -}$; (6) $^{\bullet}OH$; (7) NO (NOC-9); (8) $^1O_2$; (9) Cys. Conditions: PBS (10 mM, pH 7.4) at 25 °C; λ_{ex} = 488 nm; λ_{em} = 620 nm; Slits: 5/10 nm; voltage: 700 V.

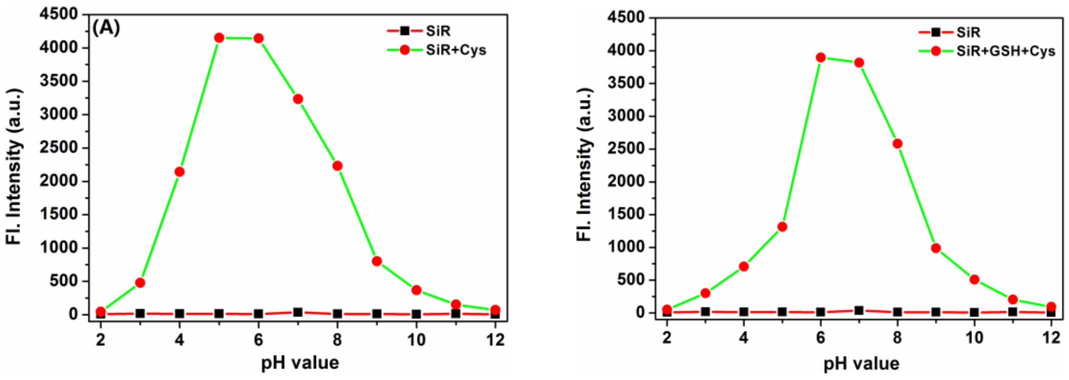

Figure S4. Fluorescence intensities of **SiR** (2.0 μM) treated with 10 equiv of Cys in the absence (A) and presence (B) of 1 mM GSH at different pH values. Condition: B-R buffer (20 mM, pH = 2–12). λ_{ex} = 488 nm; λ_{em} = 620 nm; Slits: 5/10 nm; voltage: 700 V.

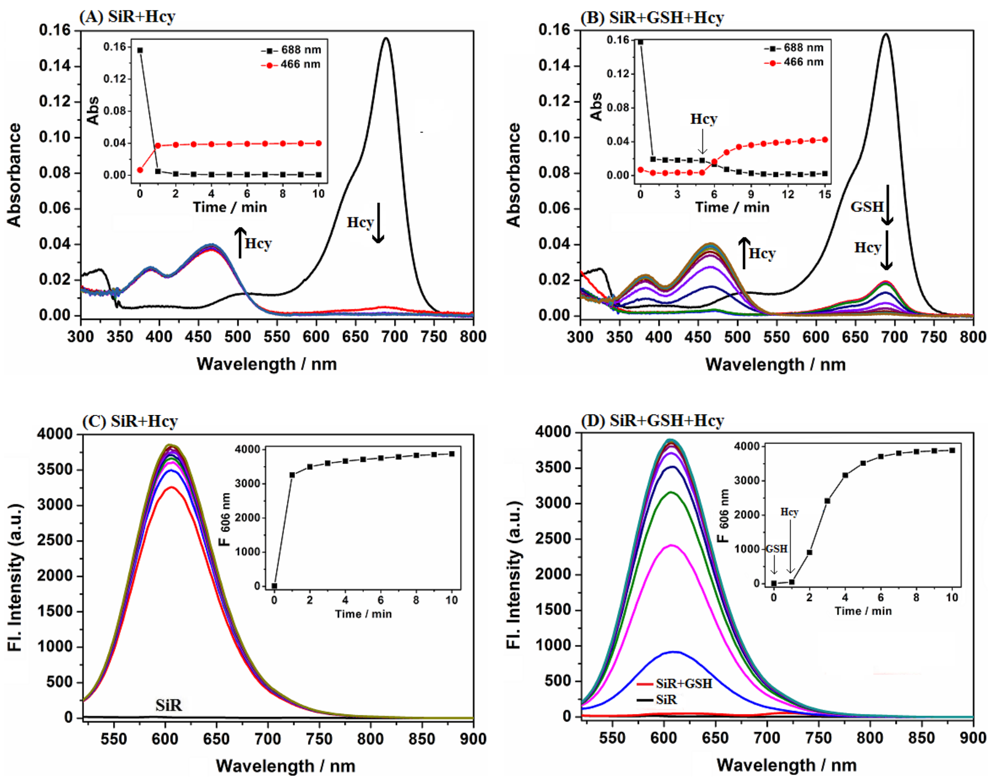

Figure S5. (A,B) Absorption spectra changes of **SiR** (2 μM) treated with Hcy (20 μM) or pretreated with GSH (1 mM) for 5 min and then treated with Hcy (20 μM) for 10 min. (C,D) Time-dependent fluorescence spectra changes of **SiR** (2 μM) treated with Hcy (20 μM) for 10 min or pretreated with GSH (1 mM) for 1 min and then treated with Hcy (20 μM) for 9 min. Spectra were recorded with the time interval of 1 min. Conditions: PBS (10 mM, pH 7.4); λ_{ex} = 488 nm and λ_{em} = 606 nm; voltage: 700 V.

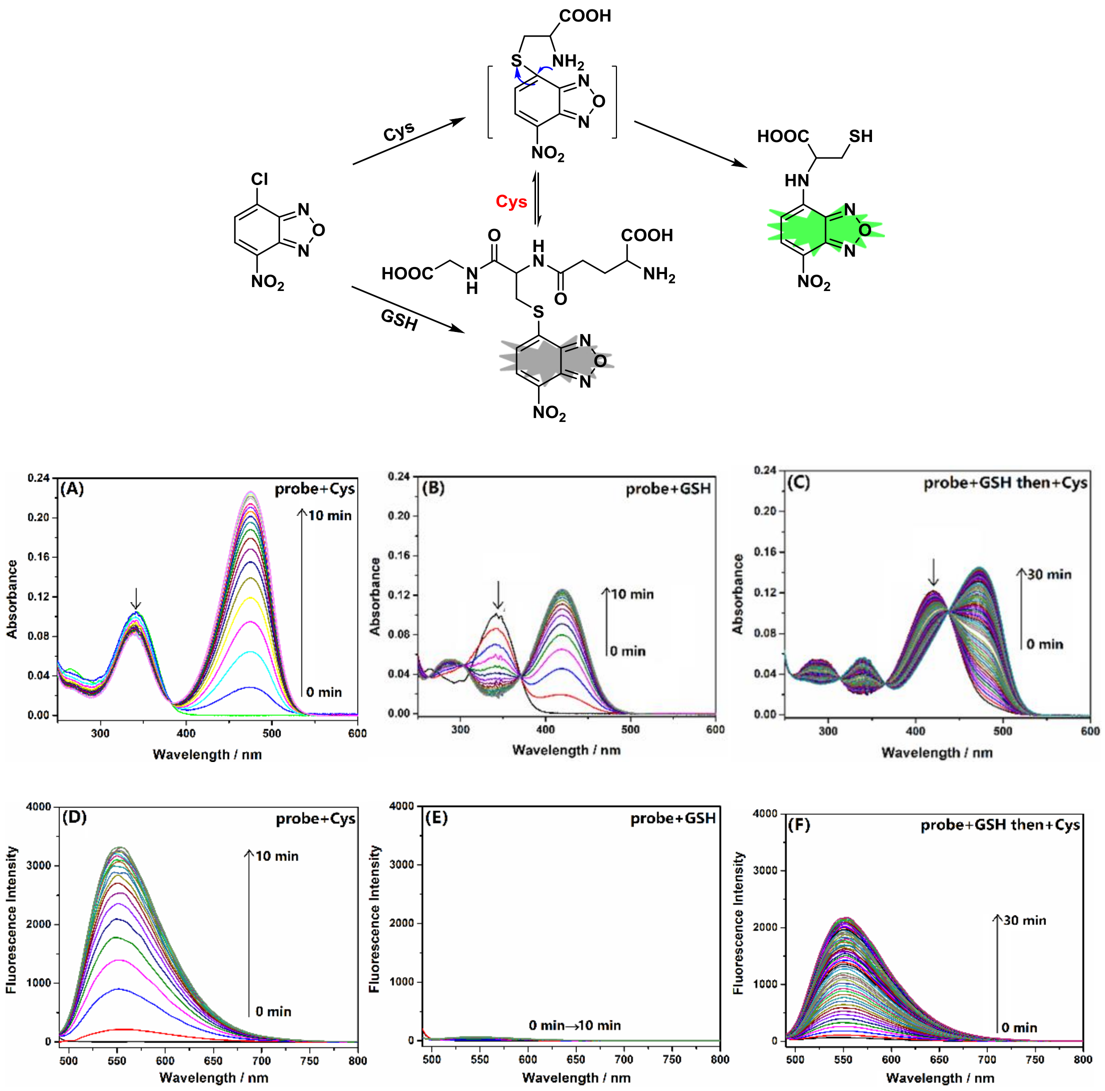

Figure S6. (A,D) Time-dependent absorption and fluorescence spectra of **NBD-Cl** (10 μM) treated with Cys (200 μM) for 10 min. (B,E) Time-dependent absorption and fluorescence spectra of **NBD-Cl** (10 μM) treated with GSH (1 mM) for 10 min. (C,F) Time-dependent absorption and fluorescence spectra of **NBD-Cl** (10 μM) pretreated with GSH (1 mM) for 10 min and then treated with Cys (200 μM) for 30 min. Spectrum was recorded every 0.5 minute. Conditions: acetonitrile–HEPES buffer (1:3, v/v, 20 mM, pH 7.4) at 25 ^{o}C. λ_{ex}: 476 nm; Slits: 5/10 nm; voltage: 600 V.

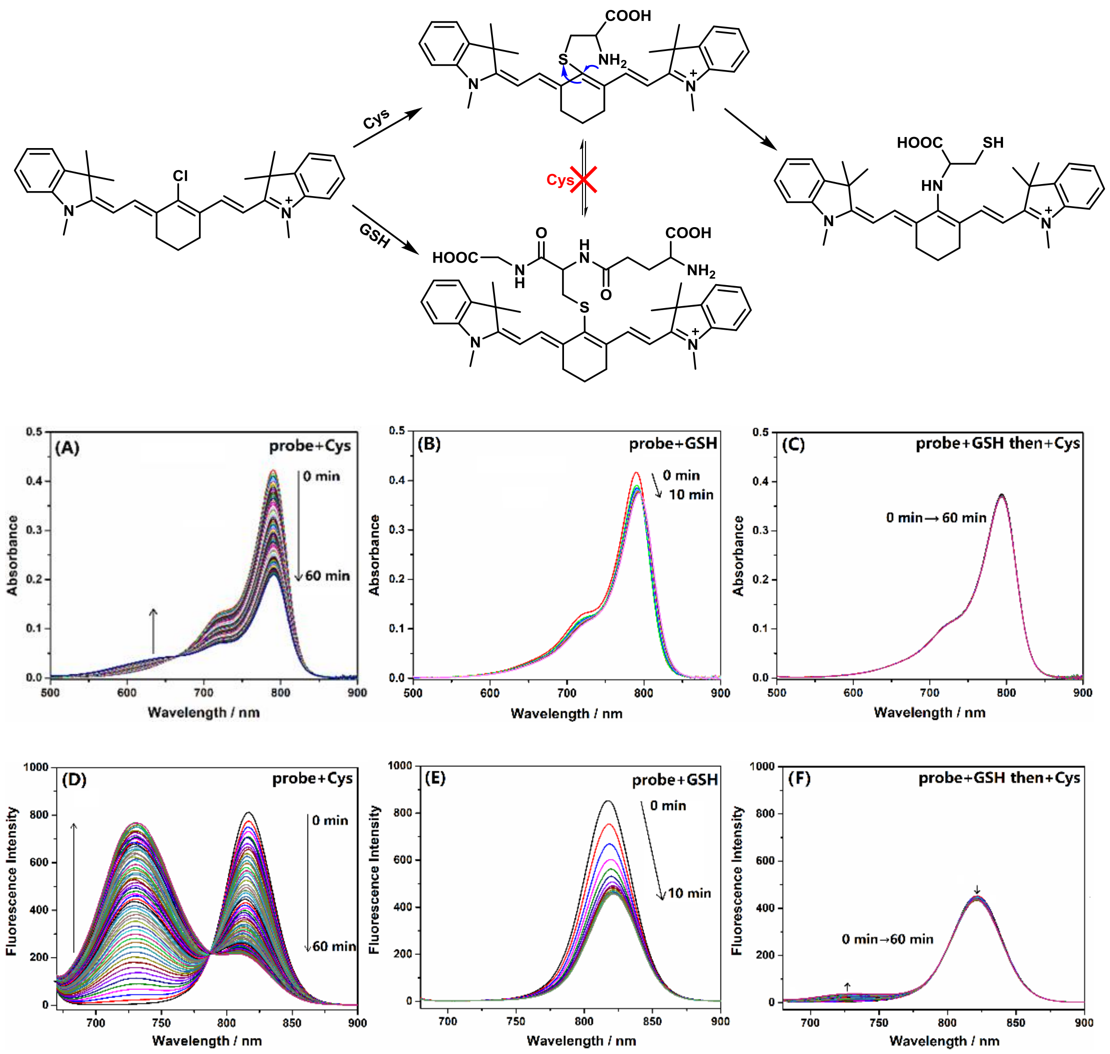

Figure S7. (A,D) Time-dependent absorption and fluorescence spectra of **Cy7-Cl** (2 μM) treated with Cys (200 μM) for 60 min. Spectrum was recorded every minute. (B,E) Time-dependent absorption and fluorescence spectra of **Cy7-Cl** (2 μM) treated with GSH (1 mM) for 10 min. Spectrum was recorded every 0.5 minute. (C,F) Time-dependent absorption and fluorescence spectra of **Cy7-Cl** (2 μM) pretreated with GSH (1 mM) for 10 min and then treated with Cys (200 μM) for 60 min. Spectrum was recorded every minute. Conditions: PBS buffer (10 mM, pH 7.4, containing 1 mM CTAB) at 25 ^{o}C. λ_{ex}: 650 nm; Slits: 10/10; voltage:700 V.

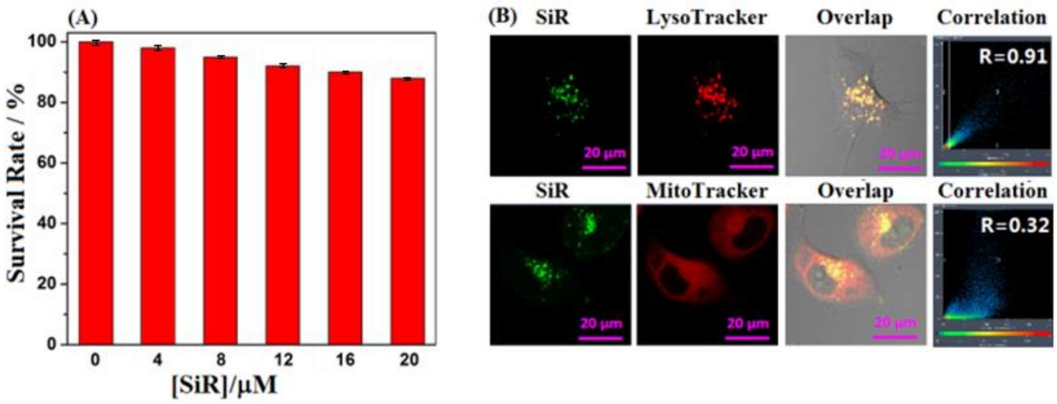

Figure S8. (A) Survival rate of HeLa cells treated with **SiR** (0–20 μM), determined by CCK8 assays. Six replicate wells were used for each control and test concentration, and error bars represent standard deviations. (B) Colocalization assay of **SiR** (2 μM) and LysoTracker Deep Red (0.5 μM) or MitoTracker Deep Red FM (0.5 μM) indicated the preferential lysosomal accumulation of the reaction product of **SiR** with intracellular Cys, i.e., **ASiR**. For **SiR**, emission was collected at 530–630 nm (λ_{ex} = 488 nm), and for LysoTracker and MitoTracker, at 650–750 nm (λ_{ex} = 633 nm). Scale bar: 20 μm.

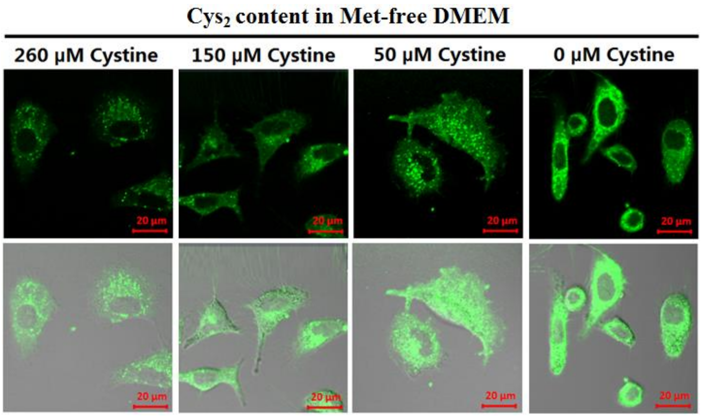

Figure S9. Confocal fluorescence images of A549 cells cultured in Met-free DMEM containing the decreased Cys_2 content for 2 days, and then treated with **SiR** (4 μM, 15 min) in PBS.Emission collected at 550–650 nm (λ_{ex} = 488 nm). Scale bar: 20 μm.

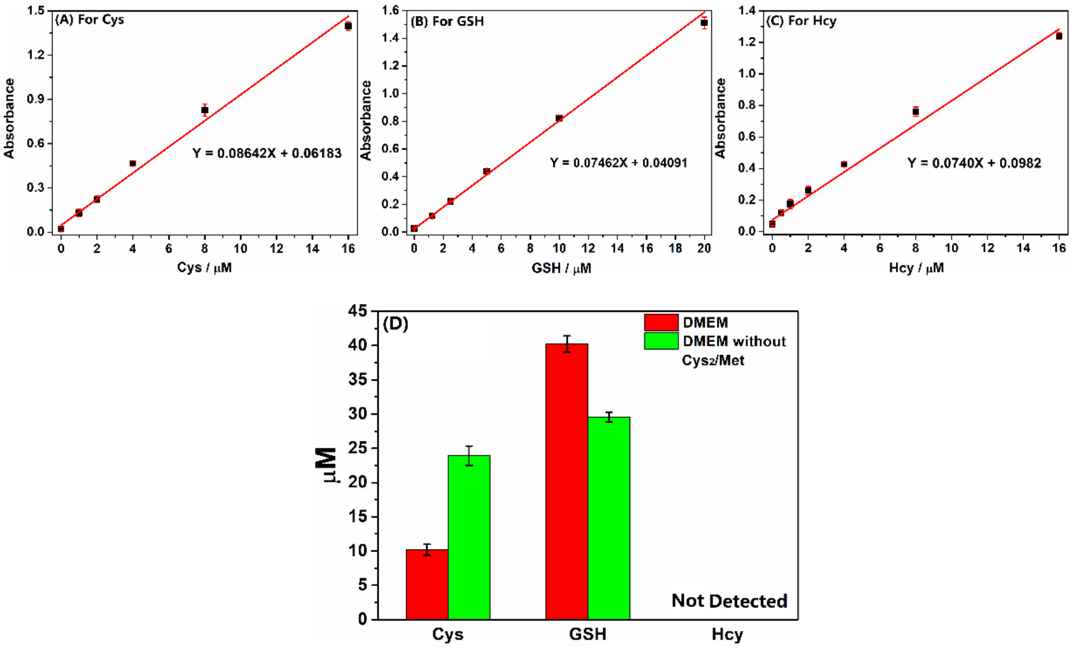

Figure S10. Detection of Cys/GSH/Hcy levels in the lysates of HeLa cells (1×10^6 cells/mL) incubated in standard and in Met/Cys_2-free DMEM medium for 2 days, respectively, using enzyme-linked immune response (ELISA) kit. (A-C) Standard curves of optical density (450 nm) versus concentrations of Cys (A), GSH (B), and Hcy (C). (D) Comparison of Cys, GSH, and Hcy levels in the lysates of HeLa cells incubated in normal and in Met/Cys_2-free DMEM mediums for 2 days, respectively.

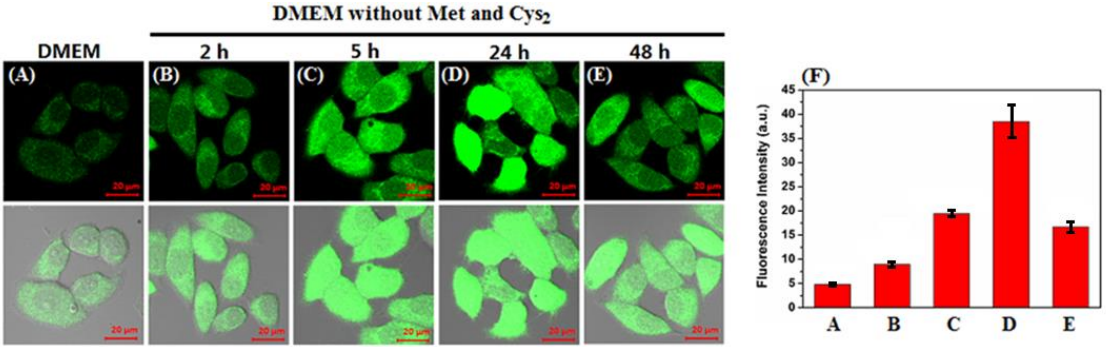

Figure S11. (A-E) Fluorescence images of HeLa cells cultured in Met/Cys_2-free DMEM for 2 h, 5 h, 24 h, and 48 h, respectively, and then treated with 2',7'-dichlorofluorescein diacetate (DCF-DA) (10 μM, 30 min) in PBS. (F) The average fluorescence intensities in (A-E). Results are statistical analyses of >10 cells, and error bars represent standard deviations. Emission was collected at 500–600 nm (excited at 488 nm).

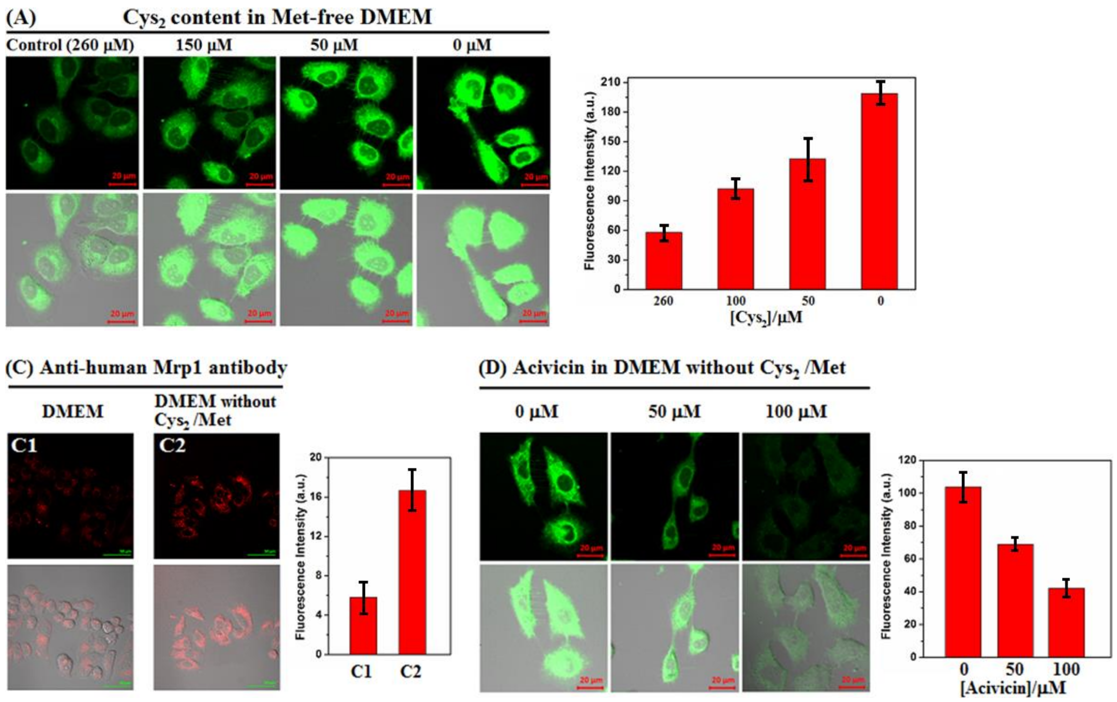

Figure S12. Fluorescence images and average fluorescence intensities in Fig. 4A, 4C, and 4D in text. Results are statistical analyses of >10 cells, and error bars represent standard deviations.

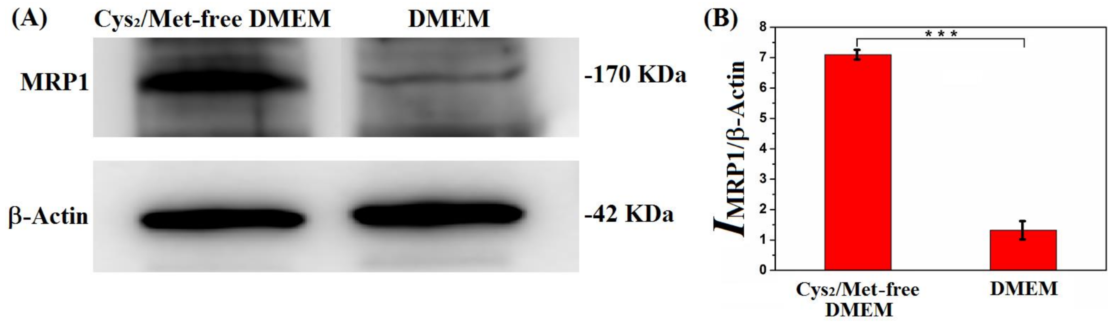

Figure S13. Mrp1 protein expression in HeLa cells. (A) Total cell extracts from HeLa cells incubated in normal and Met/Cys_2-free DMEM for 2 days, respectively, were resolved on SDS-PAGE, and the expression of Mrp1 and β-actin were determined by Western blotting. (B) The strip grayscale ratio value of MRP1 and β-Actin in (A) analyzed by image Lab 6.0 software.

13. ^{1}H NMR, ^{13}C NMR, and HRMS charts

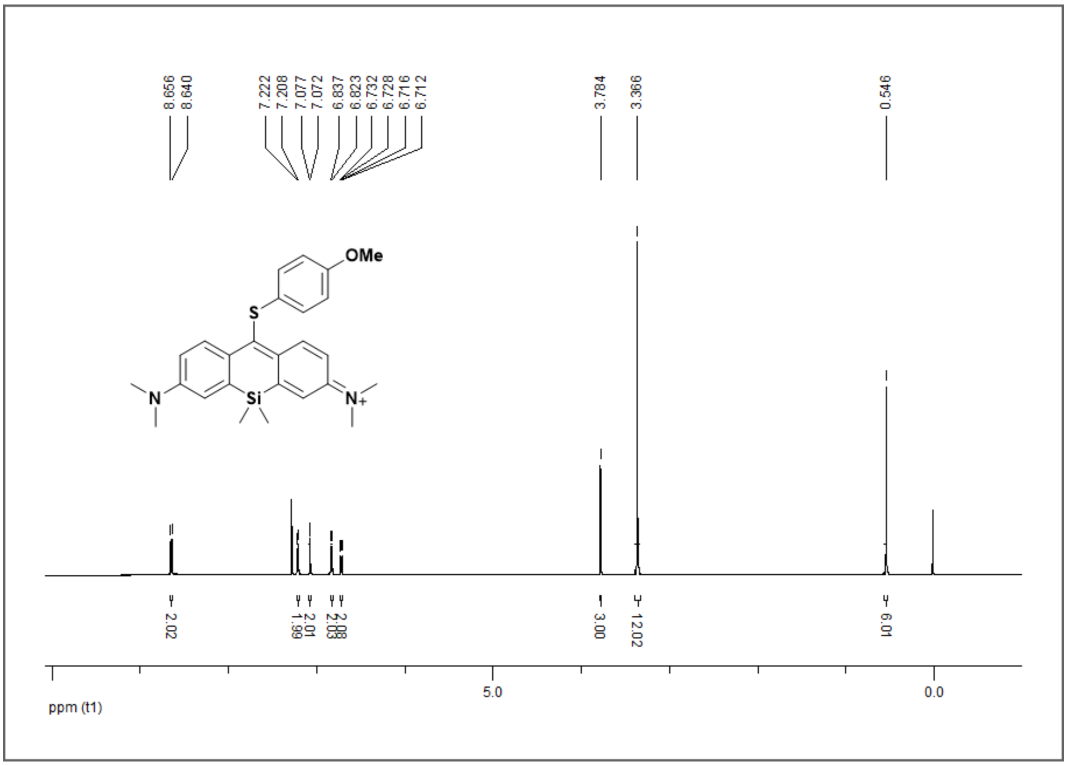

Figure S14. ^{1}H NMR chart of **SiR**(600 MHz, $CDCl_3$).

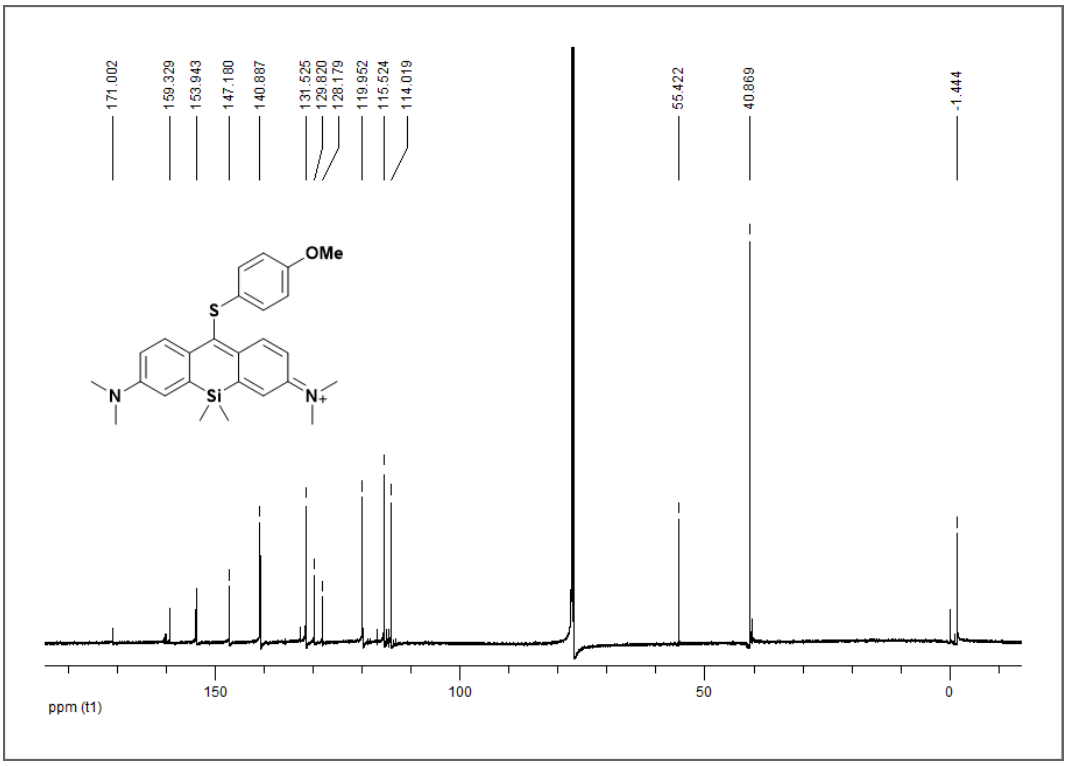

Figure S15. ^{13}C NMR chart of **SiR**(150MHz, $CDCl_3$).

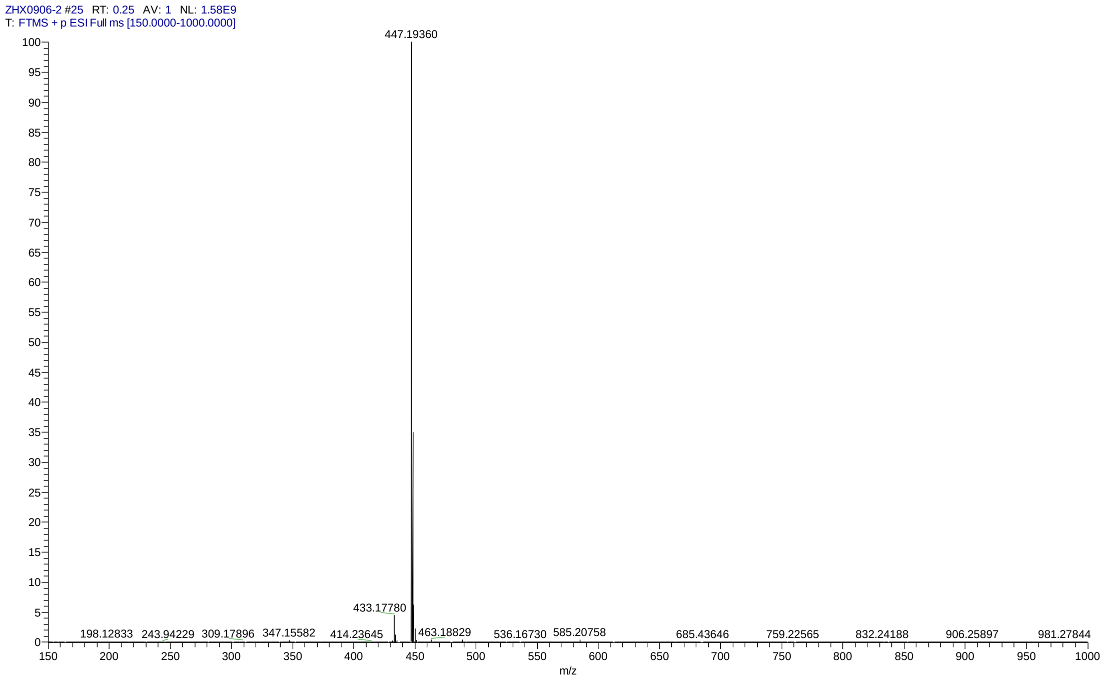

Figure S16.HRMS chart of**SiR**.

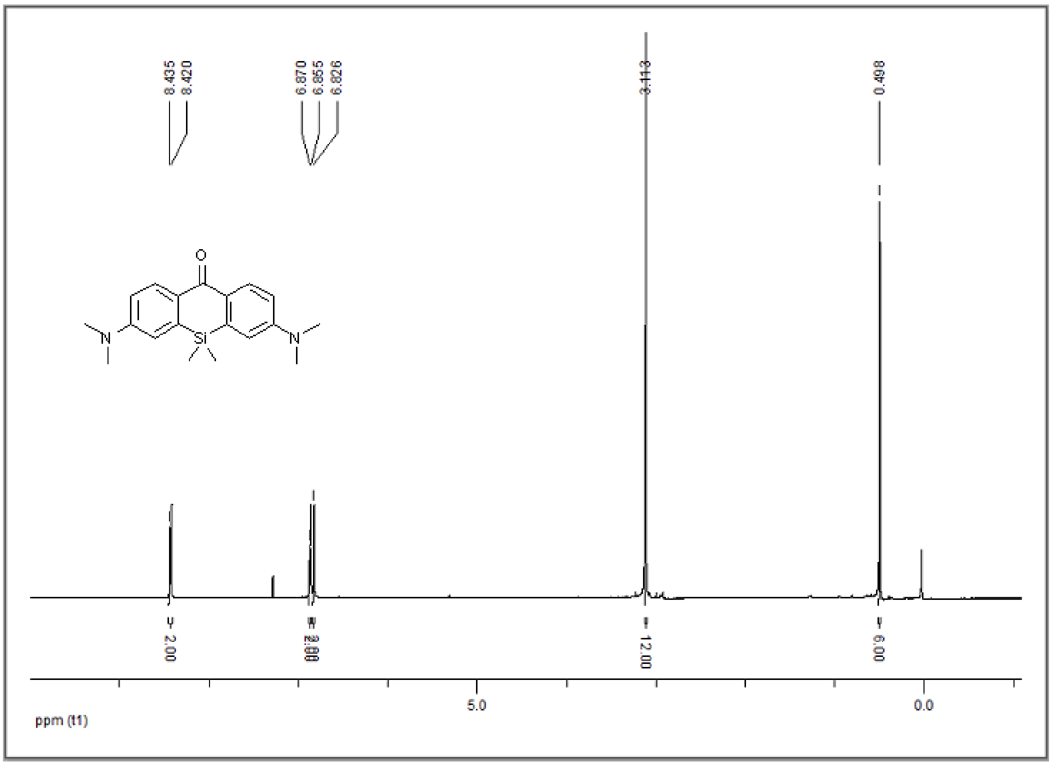

Figure S17.^{1}H NMR chart of compound **3**(600 MHz,CD_3Cl).

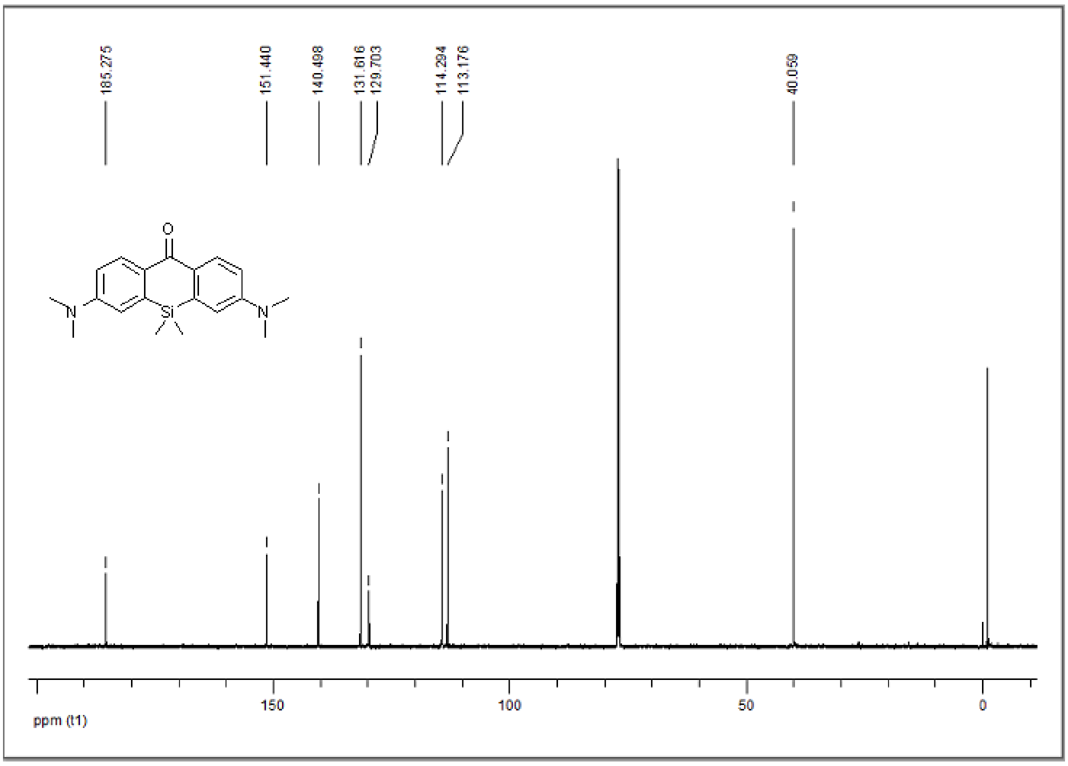

Figure S18.^{13}C NMR chart of compound **3**(150MHz,CD_3Cl).

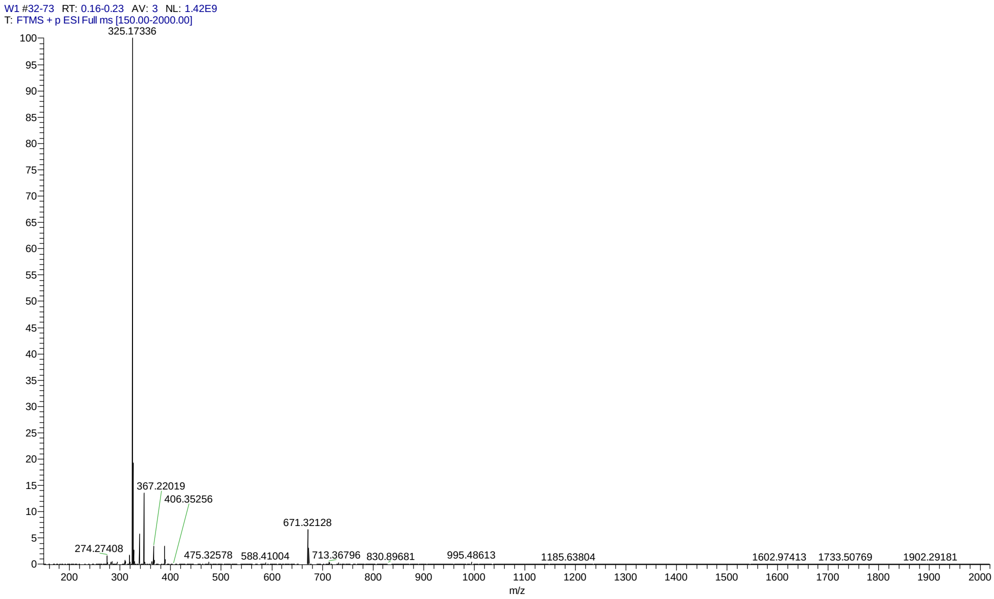

Figure S19. HRMS chart of compound **3**.

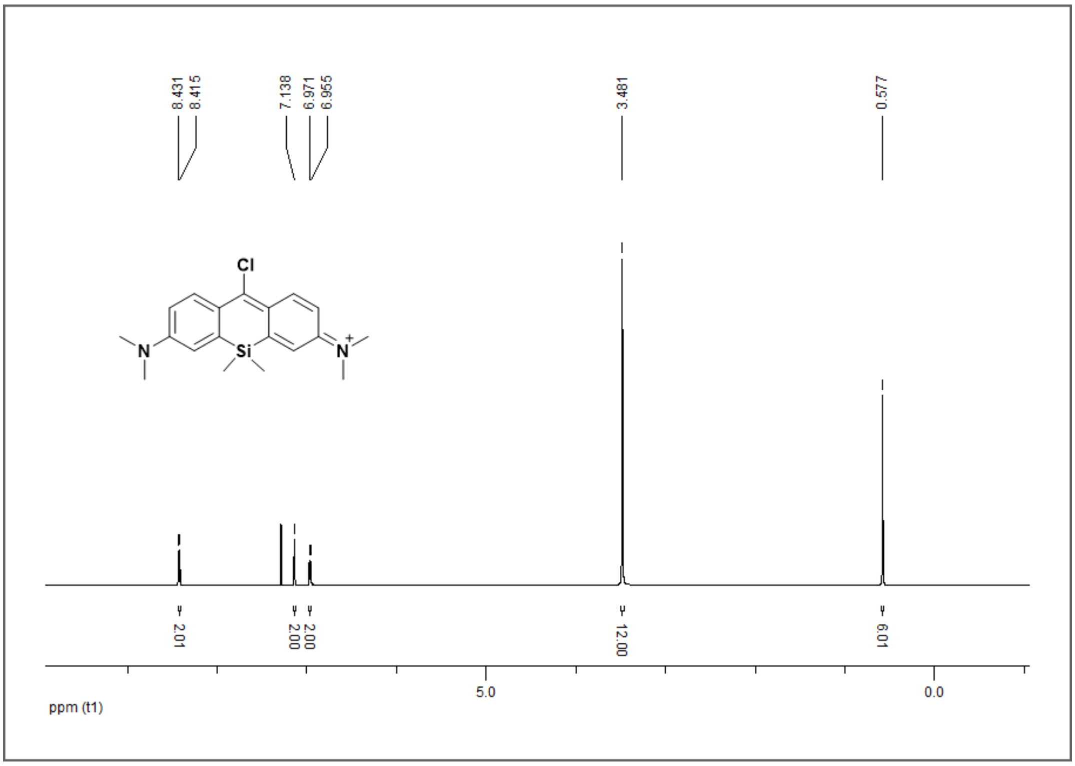

Figure S20. ^{1}H NMR chart of compound **2** (600 MHz, CD_3Cl).

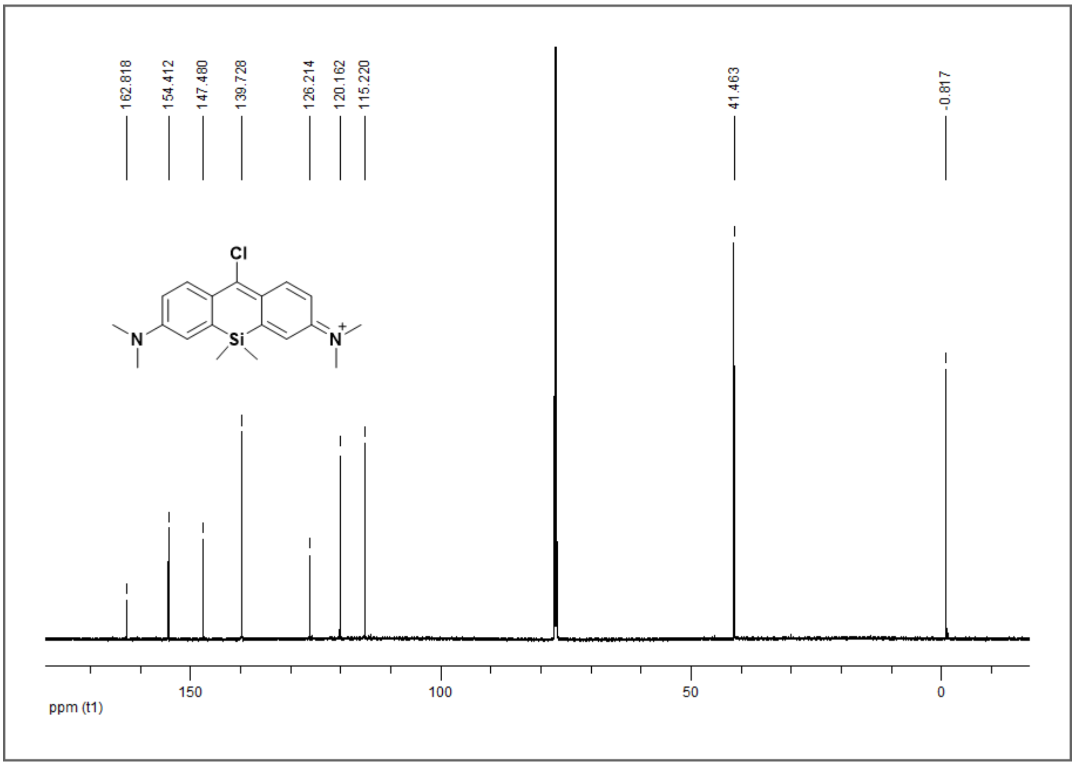

Figure S21. ^{13}C NMR chart of compound **2** (150MHz, CD_3Cl).

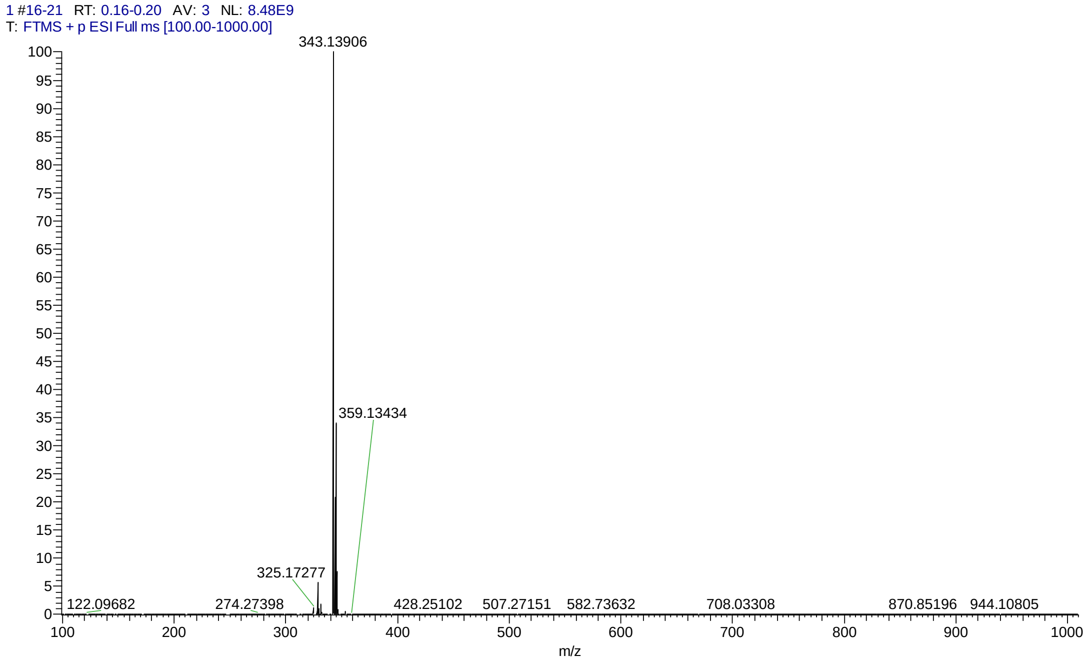

Figure S22. HRMS chart of compound **2**.

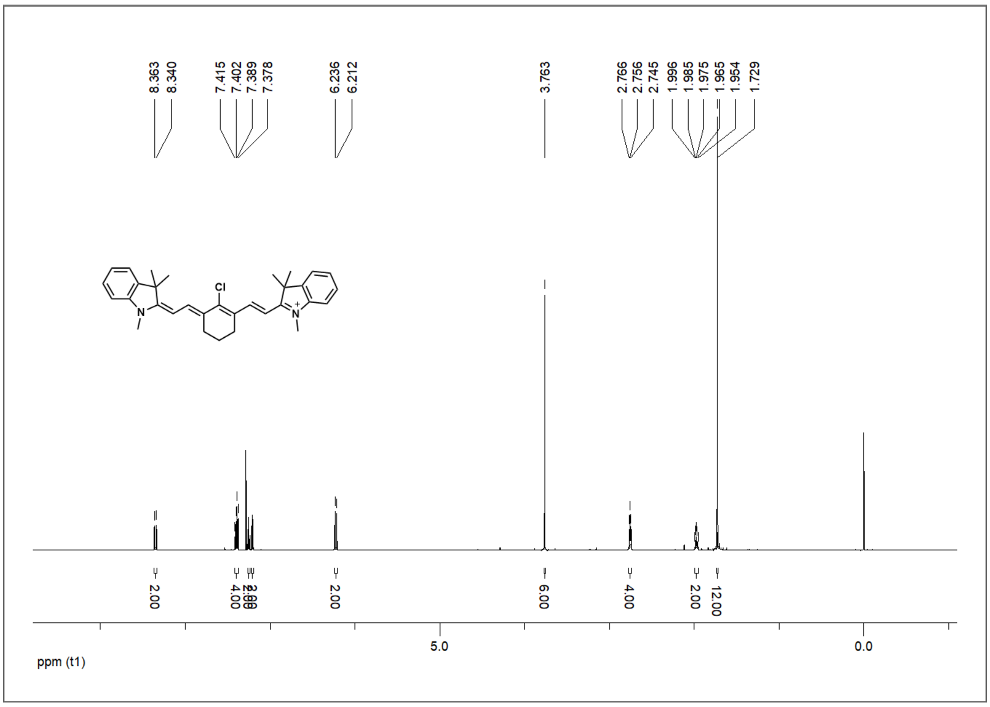

Figure S23. ^{1}H NMR chart of **Cy7-Cl**.

Reference

1 U. S. Dinish, Z. Song, C. J. H. Ho, G. Balasundaram, A. B. Ebrahim Attia, X. Lu, B. Z. Tang, B. Liu and M. Olivo. *Advanced Functional Materials*, 2015, 25(15), 2316-2325.
